# Division of Labor between PCNA Loaders in DNA Replication and Sister Chromatid Cohesion Establishment

**DOI:** 10.1016/j.molcel.2020.03.017

**Published:** 2020-05-21

**Authors:** Hon Wing Liu, Céline Bouchoux, Mélanie Panarotto, Yasutaka Kakui, Harshil Patel, Frank Uhlmann

**Affiliations:** 1 Chromosome Segregation Laboratory, The Francis Crick Institute, 1 Midland Road, London NW1 1AT, UK; 2 Bioinformatics and Biostatistics Science Technology Platform, The Francis Crick Institute, 1 Midland Road, London NW1 1AT, UK

**Keywords:** Chromosome Segregation, Sister Chromatid Cohesion, Cohesion Establishment, DNA Replication, Replication Factor C, Rfc1, Ctf18, PCNA, Eco1, *S. cerevisiae*

## Abstract

Concomitant with DNA replication, the chromosomal cohesin complex establishes cohesion between newly replicated sister chromatids. Several replication-fork-associated “cohesion establishment factors,” including the multifunctional Ctf18-RFC complex, aid this process in as yet unknown ways. Here, we show that Ctf18-RFC’s role in sister chromatid cohesion correlates with PCNA loading but is separable from its role in the replication checkpoint. Ctf18-RFC loads PCNA with a slight preference for the leading strand, which is dispensable for DNA replication. Conversely, the canonical Rfc1-RFC complex preferentially loads PCNA onto the lagging strand, which is crucial for DNA replication but dispensable for sister chromatid cohesion. The downstream effector of Ctf18-RFC is cohesin acetylation, which we place toward a late step during replication maturation. Our results suggest that Ctf18-RFC enriches and balances PCNA levels at the replication fork, beyond the needs of DNA replication, to promote establishment of sister chromatid cohesion and possibly other post-replicative processes.

## Highlights

• Distinct roles of Ctf18- and Rfc1-RFC in cohesion establishment and DNA replication
• Ctf18-RFC balances PCNA levels between leading and lagging strands
• PCNA levels correlate with cohesion establishment
• PCNA recruits Eco1 toward a late step during replication maturation

## Graphical Abstract

**Figure:**
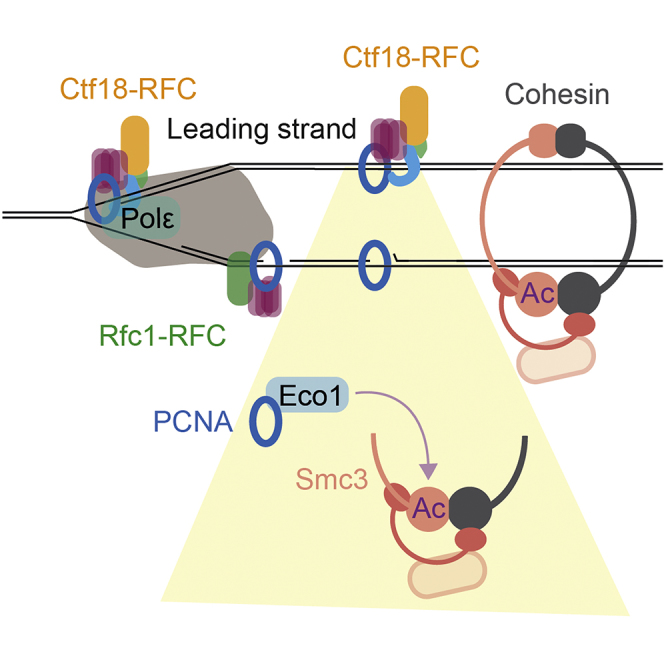

## Teaser

The PCNA sliding clamp plays multiple roles during DNA replication. Here, Liu et al. report that Ctf18-RFC supplies additional PCNA at replication forks, beyond the needs of DNA replication, balancing PCNA levels between leading and lagging strands. This serves an important role during the establishment of sister chromatid cohesion.

## Introduction

The physical pairing of two replicated genomic DNA molecules, known as sister chromatid cohesion, is essential for faithful segregation of genetic information to daughter cells. Sister chromatid cohesion is mediated by the chromosomal cohesin complex, a large proteinaceous ring formed of two coiled coil subunits, Smc1 and Smc3, a kleisin subunit Scc1, and additional HEAT repeat containing subunits Scc3 and Pds5. Topological entrapment of two sister DNA molecules by these cohesin rings forms the basis of sister chromatid cohesion (Nasmyth and Haering, 2009, Peters and Nishiyama, 2012, Uhlmann, 2016). In budding yeast, cohesin is loaded onto chromosomes in late G1 phase, which occurs at broad nucleosome-free regions with help of the Scc2-Scc4 cohesin loader complex (Muñoz et al., 2019). Cohesin translocates from these loading sites, driven by active transcription, to accumulate at sites of convergent transcriptional termination (Davidson et al., 2016, Glynn et al., 2004, Lengronne et al., 2004, Ocampo-Hafalla et al., 2016). Before DNA replication, cohesin dynamically turns over on chromosomes, its unloading facilitated by Pds5 together with its substoichiometric binding partner Wapl (Chan et al., 2012, Gerlich et al., 2006, Lopez-Serra et al., 2013, Murayama and Uhlmann, 2015).

During DNA replication, two important changes occur. First, instead of entrapping just one DNA, cohesin holds together two sister DNAs. This could be achieved if the replisome was able to replicate through cohesin rings. Alternatively, cohesin transiently loses contact with DNA and is reloaded behind the replication fork. This might occur by sequential capture of a double-stranded leading strand replication product, followed by capture of an adjacent single-stranded segment of the lagging strand. Both pathways are not mutually exclusive (Murayama et al., 2018). Second, acetylation of two conserved Smc3 lysine residues by the Eco1 cohesin acetyltransferase stabilizes cohesin’s embrace of two sister DNAs (Rolef Ben-Shahar et al., 2008, Ünal et al., 2008, Zhang et al., 2008). Smc3 acetylation stops Pds5-Wapl-dependent DNA release, thereby establishing enduring sister chromatid cohesion. Smc3 acetylation not only prevents DNA exit but also impedes further DNA entry. Therefore, the timing of Smc3 acetylation must be closely linked to sister DNA entrapment. The basis for this is not yet understood.

In addition to the essential Eco1 acetyltransferase, several non-essential replisome components contribute to the establishment of sister chromatid cohesion. These include the Ctf4 protein interaction hub that recruits the Chl1 helicase. Chl1 in turn makes direct contact with cohesin during cohesion establishment (Hanna et al., 2001, Samora et al., 2016, Skibbens, 2004). The Mrc1-Tof1-Csm3 replication progression and checkpoint mediator complex is also required for efficient cohesion establishment (Mayer et al., 2004, Xu et al., 2004). It functions in an as yet unknown capacity, as does the Ctf18-RFC complex (Mayer et al., 2001), the subject of our present study. Deficiencies in any of the above cohesion establishment factors result in compromised Smc3 acetylation (Borges et al., 2013). Therefore, all cohesion establishment factors could jointly regulate the cohesin acetylation reaction. Alternatively, cohesion establishment factors could act in various ways to facilitate sister chromatid entrapment, which in turn could be a pre-requisite for cohesin acetylation. Insight into their molecular mechanisms will be required to understand how sister chromatid cohesion is established during DNA replication.

Ctf18-RFC is a member of the Replication Factor-C (RFC) family, protein complexes that load and unload proliferating cell nuclear antigen (PCNA) sliding clamps. PCNA is involved in a plethora of DNA transactions, notably as a processivity factor for DNA polymerases but also as a docking platform for a variety of other nucleic acid processing enzymes (Ohashi and Tsurimoto, 2017). RFCs are pentameric AAA^+^ ATPases. Four small subunits, Rfc2 to Rfc5, are common to all RFCs. One of the large subunit paralogs, Rfc1, Elg1, or Ctf18, gives the complexes their names and identities (an additional Rad24 large subunit forms an RFC complex that functions with a specialized DNA damage checkpoint sliding clamp). Rfc1-RFC is the best studied and the only essential RFC complex. Its binding to PCNA wrenches open one of the PCNA trimer interfaces. Rfc1-RFC recognizes 3′ primer ends, and sequential ATP hydrolysis by its subunits releases the clamp onto DNA (Bowman et al., 2004). *In vitro*, Rfc1-RFC is able to catalyze both PCNA loading and unloading, though at replication forks its main role is thought to be PCNA loading on the lagging strand for primer elongation by DNA polymerase δ. PCNA recycling, following completion of Okazaki fragment maturation, is thought to be the task of Elg1-RFC, a specialized PCNA unloader (Kang et al., 2019, Kubota et al., 2013).

Ctf18-RFC is unique in that Ctf18 comes with two additional subunits, Ctf8 and Dcc1 (Mayer et al., 2001). Ctf18-RFC has been implicated in both loading and unloading of PCNA (Bermudez et al., 2003, Bylund and Burgers, 2005). In addition, Ctf18-RFC forms part of the DNA replication checkpoint that mediates Rad53 checkpoint kinase activation in response to replication fork stalling (Crabbé et al., 2010, Naiki et al., 2001). Further roles for Ctf18-RFC in telomere chromatin maintenance and DNA triplet repeat stability have been documented (Gellon et al., 2011, Hiraga et al., 2006). While Ctf18-RFC also recognizes primer ends, additional targeting is provided by the Dcc1 subunit that has been reported to bind both single-stranded DNA (ssDNA) and double-stranded DNA (dsDNA) as well as DNA polymerase ε (Pol ε) (Grabarczyk et al., 2018, Murakami et al., 2010, Wade et al., 2017). Dcc1 interaction with Pol ε opens the possibility that Ctf18-RFC performs at least part of its function on the leading strand (Fujisawa et al., 2017). However, whether and how any of the above properties relate to Ctf18-RFC’s role in sister chromatid cohesion is unknown.

Here we use budding yeast to investigate Ctf18-RFC’s role in cohesion establishment. We find that Ctf18-RFC loads PCNA with a slight preference for the leading strand, providing a PCNA pool that is functionally distinct from Rfc1-RFC-loaded PCNA. Experiments that interrogate Eco1’s PCNA interaction motif are consistent with a model in which Ctf18-RFC-loaded PCNA recruits the acetyl transferase to a location in the wake of the replication fork to establish sister chromatid cohesion.

## Results

### PCNA Levels Correlate with Cohesion Establishment

We previously detected Ctf18 by chromatin immunoprecipitation (ChIP) at stalled replication forks following hydroxyurea (HU) treatment (Lengronne et al., 2006), consistent with a role for Ctf18-RFC in replication checkpoint signaling. To evaluate Ctf18-RFC localization during unchallenged replication fork progression, when cohesion establishment usually takes place, we repeated Ctf18 ChIP in cells progressing synchronously through S phase following pheromone α-factor arrest and release. Figure S1A shows that Ctf18 can be seen coinciding with regions of nucleotide analog bromodeoxyuridine (BrdU) incorporation, confirming that Ctf18-RFC is present at replication forks during an undisturbed S phase.

**Figure 1:**
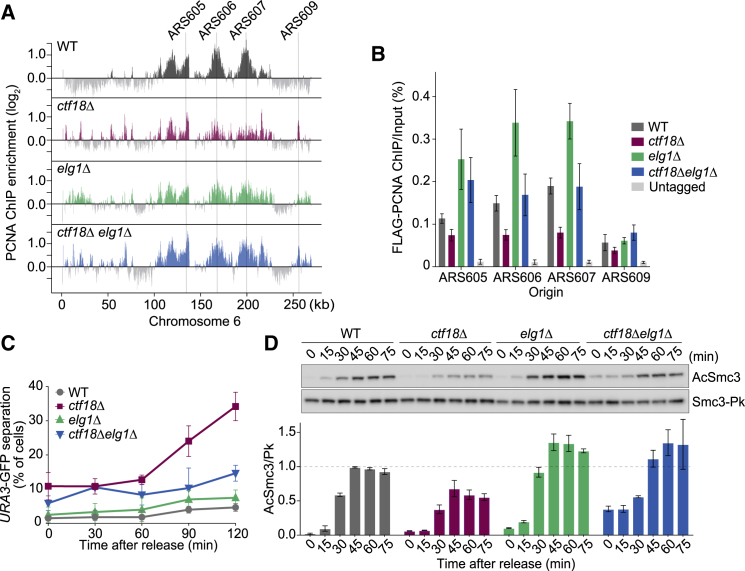
Elg1 Removal Compensates for Absence of Ctf18 (A) PCNA distributions in the absence of Ctf18 and/or Elg1. Cells were synchronized in G1 and released into HU-containing medium. PCNA chromatin immunoprecipitates were hybridized to Affymetrix GeneChip *S. cerevisiae* Tiling 1.0R arrays. Signal intensities, relative to a whole-genome DNA sample, are shown along chromosome 6. Replication origins chosen for subsequent quantitative analyses are indicated. (B) As in (A), but chromatin immunoprecipitates from N-terminally FLAG epitope-tagged PCNA were analyzed using quantitative real-time PCR using primer pairs at an early (ARS605, 606, and 607) and a late firing (ARS609) replication origin. Means ± SE from three independent experiments are shown. (C) Cells of the indicated genotypes were synchronized in G1 and released into nocodazole-containing medium to induce a mitotic arrest. Sister chromatid cohesion was assessed at the GFP-marked *URA3* locus at indicated time points. Means ± SE from three independent experiments are shown. (D) As in (C), but Smc3 acetylation was monitored by western blotting using an acetyl-Smc3-specific (AcSmc3) antibody. Total Smc3 levels were detected by its Pk epitope and served as a loading control. The AcSmc3/Smc3-Pk ratio was normalized to that in wild-type cells at 45 min. Means ± SE from three independent experiments are shown. See Figures S1A and S1B for confirmation of Ctf18 binding and PCNA loading at forks progressing through undisturbed S phase and Figures S2A–S2E for experiments separating Ctf18’s function in sister chromatid cohesion and the replication checkpoint.

Our previous results showed that PCNA levels decline at HU-stalled replication forks lacking Ctf18 (Figure 1A; Lengronne et al., 2006). We again repeated this analysis using cells progressing through synchronous S phase following α-factor arrest and release. PCNA ChIP followed by quantitative real-time PCR (ChIP-qPCR) revealed substantially decreased PCNA levels at replication forks lacking Ctf18 (Figure S1C). This suggests that Ctf18-RFC functions as a net PCNA loader both at stalled replication forks and during undisturbed replication fork progression.

To address whether Ctf18-RFC functions in sister chromatid cohesion establishment as a PCNA loader, we asked whether inactivation of the PCNA unloader Elg1-RFC can compensate for lack of Ctf18. We performed ChIP against PCNA followed by microarray analysis to visualize chromosomal distribution (Figure 1A), as well as quantitative real-time PCR to measure its levels (Figure 1B). This confirmed increased PCNA levels at replication forks in cells lacking Elg1 (Kubota et al., 2015). Notably, the PCNA reduction seen in *ctf18Δ* cells was reversed in cells lacking both Ctf18 and Elg1. PCNA levels at replication forks in *ctf18Δ elg1Δ* cells were equal or greater than in the wild-type control.

To assess the impact of PCNA levels on sister chromatid cohesion establishment, we again synchronized cells using α-factor arrest and release. Following passage through S phase, cells were arrested in mitosis by nocodazole treatment. We visualized sister chromatid cohesion of a tetO-array integrated at the *URA3* locus on chromosome 5, bound by tetR-GFP fusion proteins (Michaelis et al., 1997). As expected (Mayer et al., 2001), cells lacking Ctf18 showed a marked sister chromatid cohesion defect (Figure 1C). In contrast, cells lacking Elg1 did not show a cohesion defect when compared to a wild-type control. Strikingly, the cohesion defect of *ctf18Δ* cells was substantially reduced in cells lacking both Ctf18 and Elg1.

To analyze sister chromatid cohesion establishment in a complementary way, we used western blotting to analyze Smc3 acetylation during S phase. As previously seen, Smc3 acetylation was compromised in *ctf18Δ* cells (Figure 1D; Borges et al., 2013). In contrast, Smc3 acetylation surpassed wild-type levels in *elg1Δ* cells. Acetylation reached at least wild-type levels in cells lacking both Ctf18 and Elg1. This confirms that the cohesion defect in cells lacking Ctf18 can be rescued by additional removal of Elg1. Given the antagonistic impact of Ctf18- and Elg1-RFC on PCNA, this opens the possibility that PCNA levels at the replication fork are a limiting determinant for sister chromatid cohesion establishment. These results are consistent with and can explain the observation that *elg1Δ* partially rescues the cohesion defect in an *eco1-1* temperature sensitive strain (Maradeo and Skibbens, 2009).

### Separate Ctf18-RFC Functions in the Replication Checkpoint and in Sister Chromatid Cohesion

Ctf18-RFC functions as part of the DNA replication checkpoint, a signaling network that activates the Rad53 checkpoint kinase in response to replication fork stalling (Crabbé et al., 2010, Naiki et al., 2001). We therefore wondered whether replication checkpoint function is required for cohesion establishment. As we have seen above, sister chromatid cohesion is restored in cells lacking both Ctf18 and Elg1. In marked contrast, the sensitivity to growth on HU-containing medium is aggravated in *ctf18Δ elg1Δ* cells (Figure S2A). Rad53 phosphorylation in response to HU treatment, a sign of checkpoint activation, is mildly affected in cells lacking Ctf18 or Elg1 but almost completely abolished in cells lacking both (Figure S2B), consistent with previous reports (Bellaoui et al., 2003, Ben-Aroya et al., 2003, Kanellis et al., 2003). The fact that sister chromatid cohesion in *ctf18Δ* cells is restored by Elg1 removal but the replication checkpoint response worsens suggests that Ctf18-RFC’s sister chromatid cohesion function is distinct from its role in the replication checkpoint.

Separable roles of Ctf18-RFC in sister chromatid cohesion and the replication checkpoint also became apparent in experiments in which we analyzed the Ctf18 ATPase. Walker A motif mutations in a residue important for ATP binding (Ctf18^K189E^ [Bylund and Burgers, 2005] or Ctf18^K189A^ [Okimoto et al., 2016]) were previously used to suggest that the Ctf18 ATPase is important for the replication checkpoint and *in vitro* PCNA unloading. We confirmed the HU sensitivity of *ctf18*^K189E^ cells (Figure S2C). In contrast, the same *ctf18*^K189E^ cells were fully proficient in sister chromatid cohesion establishment (Figure S2D), thus separating the two functions.

We noticed that Ctf18^K189E^ protein levels were markedly lower than those of wild-type Ctf18 (Figure S2E), suggesting that the *ctf18*^K189E^ mutation compromises protein stability. We therefore designed two alternate ATPase mutations, a more conservative Ctf18^K189R^ change as well as Ctf18^D240A,E241A^, altering two crucial residues in the Walker B motif. Both Ctf18^K189R^ and Ctf18^D240A,E241A^ showed improved protein stability. PCNA levels at replication forks were only mildly reduced in *ctf18*^K189R^ cells when compared to *ctf18Δ* cells (Figure S2F). Sister chromatid cohesion was intact in *ctf18*^K189R^ and *ctf18*^D240A,E241A^ cells, and they showed HU-resistant growth (Figures S2C and S2D). The HU sensitivity seen in *ctf18*^K189E^ cells was therefore likely due to reduced Ctf18 stability. The replication checkpoint might be more sensitive to Ctf18-RFC levels as compared to cohesion establishment. These results also suggest that the Ctf18-RFC complex retains functionality even if its large subunit is unable to hydrolyze ATP, similar to what is seen with Rfc1-RFC (Schmidt et al., 2001).

### Ctf18-RFC-Loaded PCNA and Cohesion Establishment

**Figure 2:**
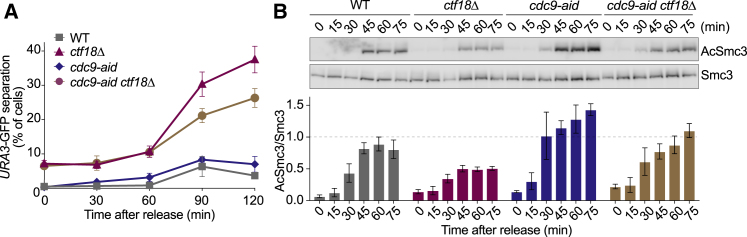
Lagging Strand PCNA Retention Facilitates Cohesion Establishment (A) Cells of the indicated genotypes were synchronized in G1 and treated with auxin for 2 h to deplete Cdc9 before release into nocodazole-containing medium. Sister chromatid cohesion was assessed at the GFP-marked *URA3* locus. Means ± SE from three independent experiments are shown. (B) As in (A) but Smc3 acetylation was quantified relative to total Smc3 levels. Means ± SE from three independent experiments are shown. See Figure S3 for further analyses on the role of PCNA in cohesion establishment.

We have to consider possible indirect explanations for cohesion restoration in *ctf18Δ* cells by Elg1 removal. Cells lacking Elg1 display DNA damage and genome instability (Bellaoui et al., 2003, Ben-Aroya et al., 2003, Johnson et al., 2016, Kanellis et al., 2003). DNA-damage-induced cohesion establishment (Ström et al., 2007, Ünal et al., 2007) might be upregulated in the absence of Elg1 and compensate for defective replication-coupled cohesion establishment. We therefore sought additional ways to test whether PCNA levels limit cohesion establishment. We took advantage of the finding that Elg1-RFC-dependent PCNA unloading requires prior Okazaki fragment ligation by the Cdc9 ligase (Kubota et al., 2015). Cdc9 depletion using an auxin-inducible degron partially restored PCNA levels at replication forks in *ctf18Δ* cells (Figure S2F). It also partially rescued sister chromatid cohesion as well as Smc3 acetylation (Figures 2A and 2B). Therefore, even in the presence of Elg1, preventing PCNA unloading by curtailing Okazaki fragment ligation improves sister chromatid cohesion.

The above experiment showed that PCNA restoration, independently of Elg1 removal, promotes sister chromatid cohesion. However, unligated Okazaki fragments following Cdc9 depletion elicit a DNA damage signal in their own right. To address more directly whether DNA-damage-induced cohesion establishment is responsible for cohesion restoration in *ctf18Δ* cells, we removed a key component of the damage-induced cohesion establishment pathway, the Chk1 checkpoint kinase (Heidinger-Pauli et al., 2008). Sister chromatid cohesion was restored in *ctf18Δ elg1Δ* cells irrespective of the presence or absence of Chk1 (Figure S2G). This suggests that DNA-damage-induced cohesion establishment was not the source of the cohesion rescue.

In another possible scenario, the absence of Ctf18-RFC leads to reduced PCNA levels, but the cohesion defect might be due to another as yet unknown function of Ctf18-RFC. Restoring PCNA by Elg1 deletion or Cdc9 depletion in turn might rescue cohesion for a reason that is unrelated to the original PCNA loss. PCNA that accumulates in the absence of Elg1 might differ from Ctf18-loaded PCNA and allow cohesion establishment via a bypass reaction. If Elg1 deletion elicits a bypass cohesion establishment reaction, such a reaction should also improve cohesion in the absence of other cohesion establishment factors. We therefore analyzed cohesion in the absence of Tof1 or Ctf4, two cohesion establishment factors that act in parallel to Ctf18 (Xu et al., 2007). *ELG1* deletion in a *tof1Δ* strain resulted in increased PCNA levels, but this did not improve sister chromatid cohesion (Figure S3A). In the case of *ctf4Δ*, sister chromatid cohesion further deteriorated in *ctf4Δ elg1Δ* cells. While we do not know the reason behind this increased cohesion defect, these results together suggest that the absence of Elg1 does not trigger a general bypass mechanism that establishes sister chromatid cohesion.

The Ctf18-RFC complex includes Ctf8 and Dcc1 subunits, which are equally important for sister chromatid cohesion as Ctf18 itself (Mayer et al., 2001; Figure S3B). If reduced PCNA levels in the absence of Ctf18 indeed caused the cohesion defect, then PCNA levels would be expected to be similarly reduced in the absence of Ctf8 or Dcc1. However, Ctf8 and Dcc1 are not required for *in vitro* PCNA loading and unloading by Ctf18-RFC (Bermudez et al., 2003, Bylund and Burgers, 2005). To test whether Ctf8 and Dcc1 are required for *in vivo* PCNA loading, we performed PCNA ChIP in strains lacking Ctf18, Ctf8, or Dcc1. We found similarly reduced PCNA levels in each case (Figure S3B). These results suggest that Ctf8 and Dcc1 are required for *in vivo* PCNA loading by Ctf18-RFC and are consistent with the idea that Ctf18-RFC acts in sister chromatid cohesion as a PCNA loader.

As a final, direct test for a role of PCNA in cohesion establishment, we introduced a PCNA trimer interface mutation, *pol30*^C81R^, that destabilizes PCNA on chromatin (Johnson et al., 2016, Lau et al., 2002). *pol30*^C81R^ cells displayed a discernable cohesion defect as well as a small but reproducible decrease in Smc3 acetylation (Figures S3C and S3D). Both could be improved by removing Elg1. This provides an additional line of evidence that PCNA directly takes part in the establishment of sister chromatid cohesion. A role for PCNA in cohesion establishment was previously suggested by cohesion defects seen in *pol30-104* cells and by their synthetic growth defect when combined with the *eco1*^ctf7-203^ allele (Moldovan et al., 2006, Skibbens et al., 1999). Our results now provide a rationale for how Ctf18-RFC acts in sister chromatid cohesion by increasing PCNA levels at replication forks.

### Rfc1-RFC Promotes DNA Replication but Not Sister Chromatid Cohesion

**Figure 3:**
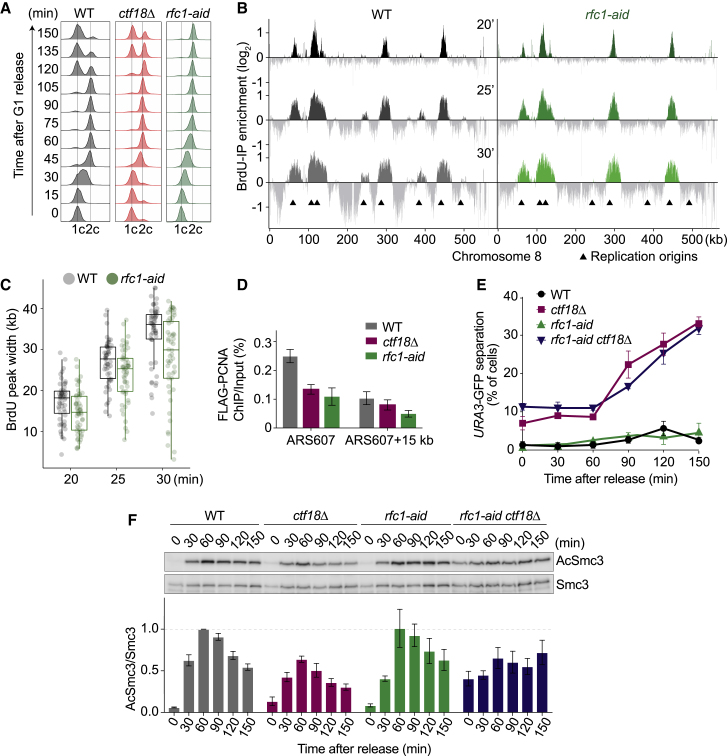
Rfc1-RFC Promotes DNA Replication but Not Sister Chromatid Cohesion (A) Cells were synchronized in G1, and Rfc1 was depleted for 2 h by auxin treatment before release into synchronous progression through S phase. DNA replication was monitored by FACS analysis of DNA content. (B) Cells were synchronized in G1 and released into BrdU-containing medium. Cells were harvested at the indicated times, and BrdU immunoprecipitates were hybridized to Affymetrix GeneChip *S. cerevisiae* Tiling 1.0R arrays. Signal intensities, relative to a whole-genome DNA sample, normalized to the median BrdU peak intensities, are shown along chromosome 8. Origin positions are indicated. (C) Boxplots of BrdU peak widths derived from (B) from 52 early origins at the indicated time points. (D) Cells were arrested in G1, and Rfc1 was depleted for 2 h by auxin treatment before release into HU-containing medium for an early S phase arrest. FLAG-PCNA chromatin immunoprecipitates were analyzed by qPCR with primer pairs around ARS607. Means ± SE from four independent experiments are shown. (E) Cells were synchronized in G1 and released into nocodazole-containing medium. Sister chromatid cohesion was assessed at the GFP-marked *URA3* locus. Means ± SE from three independent experiments are shown. (F) As in (E), but Smc3 acetylation was quantified relative to total Smc3 levels. Means ± SE from three independent experiments are shown. See Figure S4 for further characterization of the *rfc1-aid* strain.

If PCNA is a limiting factor for cohesion establishment, then the Rfc1-RFC complex might also contribute to sister chromatid cohesion by loading PCNA. To analyze this, we generated an auxin-inducible degron allele of the Rfc1 subunit, *rfc1-aid*. Rfc1 levels remained scarcely detectable following auxin addition, and cell growth was substantially impeded (Figures S4A and S4B). We synchronized cells by α-factor arrest and, following Rfc1 depletion, analyzed S phase progression by fluorescence-activated cell sorting (FACS) analysis of DNA content. Compared to wild-type or *ctf18Δ* cells, *rfc1-aid* cells showed considerably retarded DNA content duplication (Figure 3A). This is consistent with an important role of Rfc1, but less so Ctf18, during DNA replication. To investigate the reason behind slow replication following Rfc1 depletion, we analyzed BrdU incorporation into newly synthesized DNA by immunoprecipitation and microarray analysis (Figure 3B). Early origin firing following Rfc1 depletion was comparable to the wild-type control, but widening of the BrdU tracks over time was slower (Figure 3C). Rad53 was phosphorylated and late origin firing suppressed (Figures 3B and S4C), suggestive of replication checkpoint activation due to fork stalling. This suggests a key role for Rfc1-RFC during the elongation phase of DNA replication.

We next measured the impact of Rfc1 depletion on PCNA levels at replication forks. These were reduced to about half, similar to what we observed in *ctf18Δ* cells (Figure 3D). We next assessed the consequences for cohesion establishment. To our surprise, sister chromatid cohesion was unaffected by Rfc1 depletion (Figures 3E and 3F). What is more, Rfc1 depletion in a *ctf18Δ* background did not increase the cohesion or Smc3 acetylation defects, nor was DNA replication further delayed (Figures 3E, 3F and S4D). This suggests distinct roles for Ctf18- and Rfc1-RFC. While both complexes load PCNA, they appear to load different pools of PCNA. Rfc1-RFC-loaded PCNA is crucial for DNA replication but not sister chromatid cohesion. In contrast, Ctf18-RFC-loaded PCNA is dispensable for bulk DNA replication but plays a major role during cohesion establishment.

Note that this scenario changes in the absence of both Ctf18 and Elg1. In this case, Rfc1-RFC provides the only source of PCNA that, in the absence of Elg1-RFC, gains the ability to promote cohesion establishment. We will discuss this further below.

### Pol ε Interaction Is Dispensable for Ctf18-RFC Function

**Figure 4:**
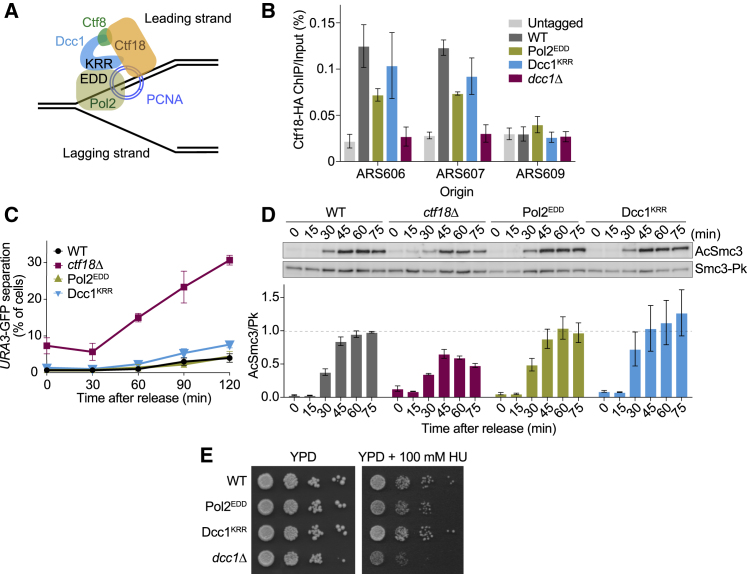
Pol ε Interaction Is Dispensable for Ctf18-RFC Function (A) Schematic of the Ctf18 interaction with the Pol ε large subunit Pol2. (B) Cells were synchronized in G1 and released into HU-containing medium for an early S phase arrest. Ctf18 enrichment close to an early (ARS606 and 607) and a late firing (ARS609) replication origin were quantified by real-time PCR. Means ± SE from three independent experiments are shown. (C) Cells were synchronized in G1 and released into nocodazole-containing medium. Sister chromatid cohesion was assessed at the GFP-marked *URA3* locus. Means ± SE from three independent experiments are shown. (D) As in (C), but Smc3 acetylation was quantified relative to total Smc3 levels. Means ± SE from three independent experiments are shown. (E) 10-fold serial dilutions of the indicated strains were spotted on Yeast extract Peptone Dextrose (YPD) agar plates without or containing 100 mM HU. See Figure S5 for further characterization of Pol2^EDD^ and Dcc1^KRR^.

Ctf18-RFC interacts via its Dcc1 subunit with the large Pol2 subunit of the leading strand DNA polymerase, Pol ε (Grabarczyk et al., 2018, Murakami et al., 2010). We therefore investigated whether Pol ε interaction is important for Ctf18 function in sister chromatid cohesion. Based on the crystal structure of Dcc1 in complex with Pol2, we introduced point mutations into Dcc1 or Pol2 that disrupt this interaction *in vitro* (Grabarczyk et al., 2018). We confirmed that these mutations, Dcc1^K364A,R367A,R380A^ and Pol2^E318A,D334A,D368A^ (short Dcc1^KRR^ and Pol2^EDD^; Figure 4A), disrupt the Ctf18-RFC interaction with Pol2 *in vivo* (Figure S5). We next used ChIP-qPCR to assess the consequence of these mutations on Ctf18-RFC recruitment to replication forks. Cells expressing Dcc1^KRR^ or Pol2^EDD^ showed reduced Ctf18-RFC levels at HU-synchronized replication forks, but Ctf18-RFC remained clearly detectable in both cases (Figure 4B). PCNA levels were reduced in Dcc1^KRR^ cells, but they remained distinctly higher than in the absence of Ctf18 (Figure S2F), consistent with the observation that Pol2 stimulates PCNA loading by Ctf18-RFC (Fujisawa et al., 2017). Together, this suggests that the Pol ε interaction contributes to but is not the only means by which Ctf18-RFC is recruited to replication forks.

We then analyzed the importance of Pol ε interaction for sister chromatid cohesion. Unlike absence of Ctf18, Dcc1^KRR^ and Pol2^EDD^ had no obvious effect on sister chromatid cohesion or Smc3 acetylation (Figures 4C and 4D). Furthermore, strains expressing Dcc1^KRR^ or Pol2^EDD^ grew on HU-containing medium almost equal to a wild-type control, unlike *dcc1Δ* cells that show pronounced HU sensitivity (Figure 4E). This suggests that Pol ε interaction is not essential for Ctf18-RFC function in sister chromatid cohesion and the replication checkpoint. Additional replisome targeting mechanisms are likely contained in the Ctf18-RFC complex, and these appear sufficient to support its main functions.

### Ctf18- and Rfc1-RFC Distribute to Leading and Lagging Strands

**Figure 5:**
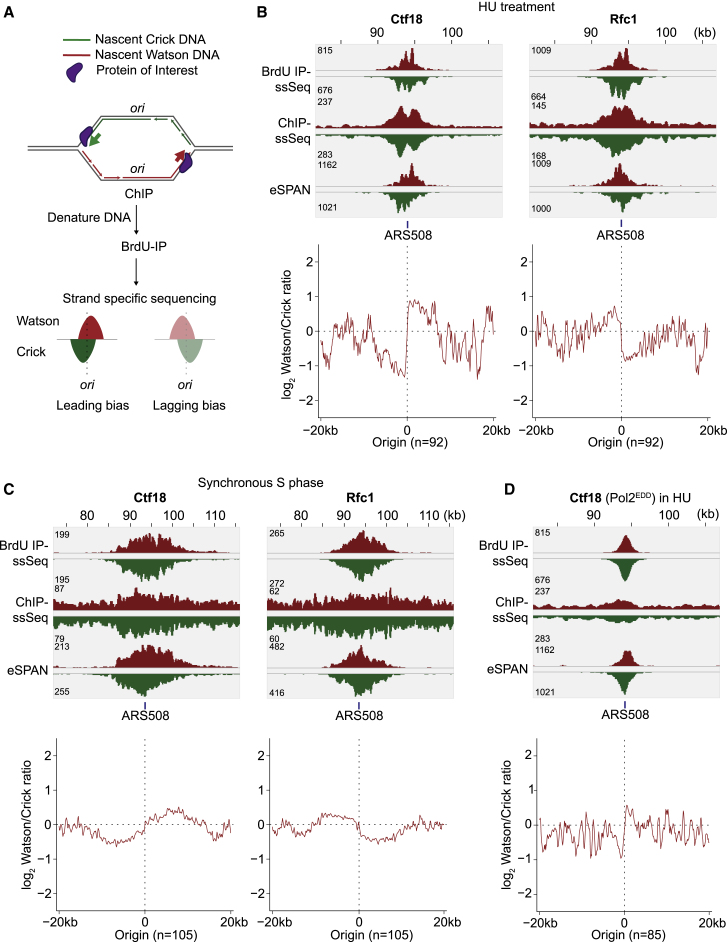
Ctf18 and Rfc1 Distribute to the Leading and Lagging Strands (A) Schematic of eSPAN, combining ChIP with strand-specific nascent DNA sequencing. (B) Cells were synchronized in G1 and released into medium containing BrdU and HU. DNA recovered by BrdU-IP, ChIP against Ctf18 or Rfc1, and ChIP followed by BrdU-IP (eSPAN) was subject to strand-specific sequencing. Watson (red) and Crick (green) reads around ARS508 are shown, as well as the averaged strand bias, normalized to BrdU reads, surrounding 92 early, well-separated origins. (C) Ctf18 and Rfc1 eSPAN analysis in cells synchronized in G1 and released into synchronous S phase progression in BrdU-containing medium for 26 min. (D) Ctf18 eSPAN analysis in Pol2^EDD^ cells synchronized in G1 and released into BrdU- and HU-containing medium. See Figure S6 for Ctf18 and Rfc1 eSPAN analyses in cells lacking their respective counterparts.

To ascertain where Ctf18- and Rfc1-RFC act at the replication fork, we utilized enrichment and sequencing of protein-associated nascent DNA (eSPAN; Yu et al., 2014). This technique uses BrdU incorporation to label nascent strands. ChIP against a protein of interest is followed by DNA denaturation and immunopurification of the BrdU-containing strand. Its location and strandedness is then determined using a strand-specific sequencing protocol. A protein that associates with leading strands will retrieve Crick strand sequences upstream and Watson strand sequences downstream of a replication origin (Figure 5A) and vice versa for a protein enriched on lagging strands. As reported, Rfc1 showed a pronounced lagging strand bias at HU-synchronized replication forks (Yu et al., 2014). In contrast, Ctf18 showed a prominent leading strand bias (Figure 5B). We repeated eSPAN using an early S phase time point of cultures traversing through an undisturbed cell cycle following α-factor synchronization (Figure 5C). This confirmed preferential distribution of Ctf18 and Rfc1 to the leading and lagging strands, respectively.

To determine whether Pol ε interaction was responsible for the Ctf18-RFC leading strand bias, we repeated Ctf18 eSPAN in the Pol2^EDD^ background. The leading strand bias was now less pronounced but still discernible (Figure 5D). We conclude that Ctf18-RFC preferentially engages with the leading strand, a preference that is augmented by but does not solely depend on its interaction with Pol ε.

### Ctf18-RFC Is Integral to Balancing PCNA Levels between Leading and Lagging Strands

**Figure 6:**
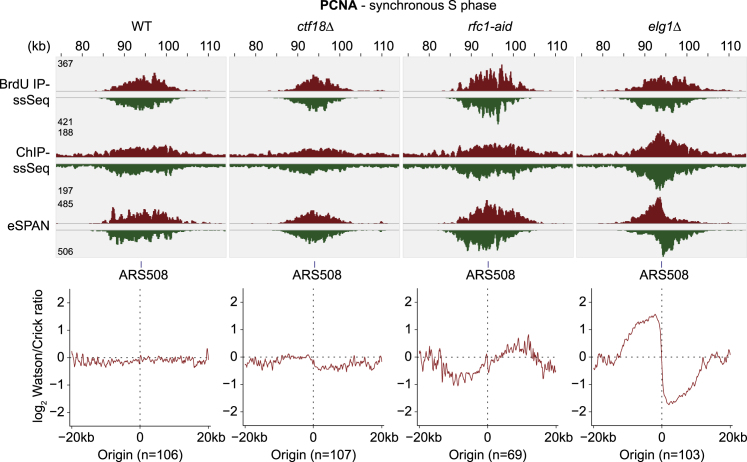
RFC Complexes Balance PCNA Levels at Replication Forks PCNA eSPAN analysis was performed in the indicated strain backgrounds as in Figure 5. Cells were synchronized in G1 and released into synchronous S phase progression in BrdU-containing medium for 26 min. Averaged strand bias, normalized to BrdU reads, surrounding early, well-separated origins is shown.

We next investigated how RFC complexes shape the PCNA distribution at replication forks. We performed PCNA eSPAN in early S phase following α-factor block and release. It was reported that PCNA is enriched on the lagging strand under these conditions, as expected from frequent PCNA loading during Okazaki fragment synthesis (Yu et al., 2014). Against these expectations, we reproducibly found no strand bias, indicating an equal PCNA distribution on both leading and lagging strands (Figure 6). Two technical differences from the prior experiment might explain this. We used an α-PCNA antibody to precipitate unmodified PCNA (Yu et al., 2014) instead of an epitope tag fusion to the PCNA C terminus, a region known to be important for PCNA function (Kelman et al., 1999). Furthermore, our experiment was performed at 25°C instead of 16°C, which might alter PCNA loading or unloading dynamics. Our results suggest that equal PCNA amounts are present on the leading and lagging strands at undisturbed DNA replication forks.

Elg1 is thought to unload PCNA after completion of Okazaki fragment synthesis. As expected (Yu et al., 2014), following Elg1 removal, PCNA became prominently enriched on lagging strands (Figure 6). In contrast, Rfc1 depletion resulted in a marked PCNA loss from the lagging strand and consequent enrichment on the leading strand. This confirms the dominant role of Rfc1-RFC in PCNA loading during Okazaki fragment synthesis. It also confirms the presence of substantial PCNA amounts on the leading strand that become exposed in the absence of Rfc1-RFC.

In *ctf18Δ* cells, PCNA gained a noticeable bias toward the lagging strand (Figure 6). This corroborates the role of Ctf18-RFC in leading strand PCNA loading. However, the lagging strand bias was relatively small, given the substantial quantitative PCNA loss from replication forks in *ctf18Δ* cells (Figure 1B). A likely explanation is that Ctf18-RFC loads PCNA onto both leading and lagging strands with a preference for the leading strand.

An alternative explanation for the small lagging strand PCNA bias in *ctf18Δ* cells is that Rfc1-RFC takes over leading strand PCNA loading. To test whether RFC complexes compensate for each other, we performed Rfc1 and Ctf18 eSPAN in each other’s absence. The Rfc1 lagging strand bias was unaltered in the absence of Ctf18, and equally the Ctf18-RFC leading strand bias was unaffected by Rfc1 depletion (Figure S6A). This makes it unlikely that Rfc1 compensates for Ctf18-RFC in leading strand PCNA loading, emphasizing the distinct functions of the two RFC complexes.

Finally, we addressed how Elg1 removal restores PCNA levels at replication forks lacking Ctf18-RFC. Elg1 removal might restore leading strand PCNA in this background. However, this was not the case. The lagging strand PCNA bias in *ctf18Δ* cells was greatly augmented in *ctf18Δ elg1Δ* cells, and it even exceeded the strong bias seen in *elg1Δ* cells (Figure S6B). This suggests that sister chromatid cohesion is rescued in *ctf18Δ elg1Δ* cells primarily by increased PCNA levels on the lagging strand.

### Ctf18-RFC Function Invokes Cohesin Acetylation

**Figure 7:**
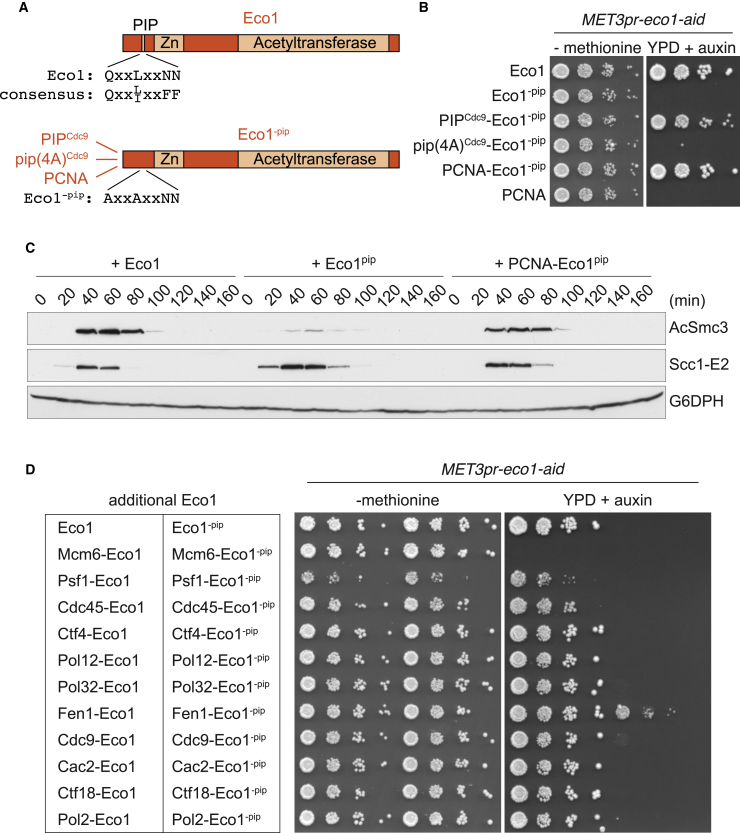
Eco1 Functions in Concert with PCNA at a Late Stage of Replication (A) Schematic of Eco1 and its PIP box, as well as the fusion constructs made with the PIP box mutant protein (Eco1^-pip^). (B) 10-fold serial dilutions of *MET3pr-eco1-aid* cultures were spotted on medium lacking methionine or on YPD medium containing auxin to repress Eco1 expression and induce its degradation. The indicated proteins were additionally expressed under control of the Eco1 or PCNA promoters, respectively. (C) *MET3pr-eco1-aid* cells expressing the indicated additional proteins were synchronized in G1, depleted of endogenous Eco1, and released into synchronous cell-cycle progression. Smc3 acetylation was analyzed by western blotting. Scc1 served as a marker for cell-cycle progression, and Glucose-6-phosphate dehydrogenase (G6PD) served as the loading control. (D) 10-fold serial dilutions of *MET3pr-eco1-aid* cultures that in addition harbored Eco1pip fusions with the indicated proteins at their respective endogenous gene loci were spotted on the indicated media. See Figure S7 for demonstration that Ctf18 targets cohesin acetylation, for an Eco1-PCNA interaction analysis, and for protein expression levels of the Eco1^-pip^ fusion proteins.

With the aim to gain further insight into Ctf18-RFC-loaded PCNA, we sought its downstream effector in sister chromatid cohesion. The Eco1 cohesin acetyltransferase has been linked to PCNA (Skibbens et al., 1999). A degenerate PCNA Interacting Peptide (PIP box) has been identified within Eco1 (Figure 7A; Moldovan et al., 2006, Song et al., 2012). However, this PIP box lacks two key aromatic residues, and reported physical interactions between Eco1 and PCNA have proven hard to reproduce. Recently, the MCM helicase was proposed as an alternative Eco1 receptor at the replisome (Ivanov et al., 2018, Minamino et al., 2018). We therefore asked whether cohesin acetylation lies downstream of Ctf18-RFC during cohesion establishment.

If the Ctf18-RFC effector is Eco1, then Ctf18 should become inconsequential in a yeast genetic background in which cohesin acetylation no longer takes place. Eco1 modifies two conserved Smc3 lysines. Cells in which both of these are replaced by either arginines or asparagines are inviable. However, a mixed arginine-asparagine mutant (*SMC3*^K112R,K113N^) allows cell proliferation in the absence of Eco1 (Borges et al., 2010). *SMC3*^K112R,K113N^ cells show compromised sister chromatid cohesion, but the presence or absence of Ctf18 no longer affected cohesion in this background (Figure S7A). Putting together the observations that cohesin acetylation is reduced in the absence of Ctf18 (Figure 1D) and that Ctf18 no longer impacts on cohesion establishment in the *SMC3*^K112R,K113N^ strain suggests that Ctf18-RFC functions upstream of cohesin acetylation. Consistent with cohesin acetylation as the functional target of Ctf18-RFC, the cohesion defect in *ctf18Δ* cells is rescued by removing the cohesin unloader Wapl that acetylation counteracts (Borges et al., 2013).

### Eco1 Functions with PCNA in the Wake of the Replication Fork

As we and others failed to detect a physical interaction between PCNA and Eco1 by conventional co-immunoprecipitation, we took an alternative approach to investigate Eco1’s PIP box. We introduced two point mutations into the degenerate PIP box QxxL motif, changing it to AxxA (Eco1^-pip^). Eco1^-pip^ failed to complement Eco1 function and elicited a pronounced sister chromatid cohesion defect (Figures 7B and S7B), consistent with earlier reports (Moldovan et al., 2006). If the Eco1 PIP box indeed mediates PCNA interaction, we should be able to replace it with the PIP box of another protein. We therefore crafted 15 amino acids from the N terminus of Cdc9 ligase, including its QxxLxxFF PIP box consensus sequence, onto the Eco1^-pip^ N terminus. This, but not fusion of an AxxAxxAA variant of the same sequence, restored cell growth and sister chromatid cohesion (Figures 7B and S7B). In a further test, we fused Eco1^-pip^ directly to PCNA. Again, this bestowed Eco1^-pip^ with the ability to support cell growth and sister chromatid cohesion. The PCNA-Eco1^-pip^ fusion also restored Smc3 acetylation, which is lost in cells expressing Eco1^-pip^ (Figure 7C). Together, these results are consistent with the possibility that Eco1’s degenerate PIP box indeed targets PCNA to promote sister chromatid cohesion.

An alternative explanation for the above observations is that PCNA-targeting bypasses the requirement for the QxxL motif that would normally mediate interaction with another protein. To study the Eco1-PCNA interaction directly, we immobilized recombinant purified His_6_-Eco1 or His_6_-Eco1^-pip^ protein on Ni^2+^ beads. Eco1, but not Eco1^-pip^, retained PCNA from cell extracts that we passed over these beads (Figure S7C). These results are most easily explained if the Eco1 PIP box indeed engages PCNA. The complex nature of these cell extracts leaves open the possibility that additional factors contribute to the interaction.

Knowing that we can restore Eco1^-pip^ protein function by fusing it to PCNA, we used a similar approach to address where at the replication fork Eco1 must act. We fused Eco1^-pip^ to a panel of additional replisome components that we selected to represent different locations at the replication fork (Figures 7D and S7D). Fusions were made to the endogenous gene loci to ensure that the fusion proteins take the place of their original targets. Of 11 fusions tested, only a Fen1-Eco1^-pip^ fusion restored robust cell growth. A much smaller degree of growth was enabled by a Cdc9-Eco1^-pip^ fusion. Fen1 is the flap endonuclease that acts during Okazaki fragment maturation. Like Cdc9, Fen1 interacts with PCNA. This suggests that Eco1 takes up a position close to PCNA during the late stages of DNA replication to function in cohesion establishment. We note that fusion of neither wild-type Eco1 nor Eco1^-pip^ to the other candidate Eco1 receptor, the MCM helicase (Ivanov et al., 2018, Minamino et al., 2018), supported cell growth in our assay.

## Discussion

Ctf18-RFC was long known to play an important role in the establishment of sister chromatid cohesion. It had been implicated in PCNA loading and unloading and in the replication checkpoint, among other functions. If forks were to transiently stall when they meet cohesin, the replication checkpoint might come into play during sister chromatid cohesion establishment. This idea is supported by the fact that the replication checkpoint mediators Mrc1, Tof1, and Csm3 also contribute to cohesion establishment. However, our molecular dissection suggests that Ctf18-RFC acts in sister chromatid cohesin separately from its checkpoint role, most likely by loading PCNA. We find that Eco1 is the likely PCNA downstream effector and we narrow down its place of action in budding yeast to a late stage during DNA replication.

### Ctf18-RFC Enriches and Balances PCNA at Replication Forks

Our analyses revealed that PCNA is evenly distributed between leading and lagging strands at DNA replication forks. This came as a surprise because PCNA is thought to function predominantly during lagging strand synthesis. Approximately every 150 base pairs, a new Okazaki fragment primer is laid down that is elongated with the help of Rfc1-RFC, PCNA, and Pol δ. Leading strand synthesis by Pol ε is also facilitated by PCNA but is thought to proceed processively over much longer distances. It has been suggested that Pol ε stalling triggers new rounds of PCNA loading by Ctf18-RFC on the leading strand (Fujisawa et al., 2017), but the frequency with which such events happen is unknown.

While lagging strand replication is therefore likely to utilize a greater number of PCNA molecules compared to the leading strand, two mechanisms emerge that balance PCNA levels between the two strands. First, Elg1-RFC unloads PCNA from the lagging strand following completion of Okazaki fragment maturation. Second, we find that Ctf18-RFC adds substantial amounts of PCNA onto the leading strand. While possible consequences of leading strand PCNA for DNA replication remain to be investigated, an important role might lie in processes additional to DNA replication. Given the inability of Ctf18 to support DNA replication, we imagine that Ctf18-RFC loads PCNA away from sites of DNA synthesis, possibly in a post-replicative fashion. This could increase PCNA levels that persist following completion of DNA replication both on the leading and lagging strands.

Without Ctf18, PCNA levels on the leading strand decline more than those on the lagging strand. However, cohesion can be restored by removing Elg1-RFC, which causes PCNA accumulation on the lagging strand. This is at first sight paradoxical but could be explained if PCNA on both strands can support cohesin acetylation and cohesion establishment as long as PCNA is present at an appropriate distance from the fork. This idea is supported by the observation that targeting Eco1 to Fen1, which acts late during Okazaki fragment maturation, supports cohesion establishment. Note that cells lacking Elg1 show genome instability due to excessive PCNA retention (Johnson et al., 2016). Therefore, lower but balanced PCNA retention on both leading and lagging strands might provide a favorable opportunity for cohesion establishment.

### Distinct Rfc1-RFC, Ctf18-RFC, and Elg1-RFC Specificities

What gives the three eukaryotic RFC complexes, Rfc1-, Ctf18-, and Elg1-RFC, their specificities? The biochemical characterization of Rfc1- and Ctf18-RFC showed that both complexes recognize primer ends as a substrate for PCNA loading (Bermudez et al., 2003, Bylund and Burgers, 2005). Rfc1-RFC, but not Ctf18-RFC, is additionally able to load PCNA onto a nicked double-stranded DNA template. Despite these principal similarities, strict distinctions are encountered *in vivo*. There, Ctf18-RFC seems unable to recognize primer ends to support DNA synthesis. Rfc1-RFC in turn appears unsuited to load PCNA for use in cohesion establishment. The Ctf18-RFC complex includes two additional subunits, Ctf8 and Dcc1, that distinguish it from Rfc1-RFC. These subunits link Ctf18-RFC to the leading strand DNA polymerase ε. Cell fractionation experiments have suggested that chromatin recruitment of Ctf18-RFC during S phase is also helped by Ctf4 (Lengronne et al., 2006). Whether Ctf18-RFC directly interacts with the Ctf4 replisome interaction hub and whether this additionally contributes to Ctf18-RFC function remains to be investigated.

PCNA unloading by Elg1-RFC occurs after completion of lagging strand synthesis by Okazaki fragment ligation. *In vitro*, Elg1-RFC unloads PCNA at primer ends, at nicks, or from covalently closed dsDNA (Kang et al., 2019, Kubota et al., 2015). How unloading *in vivo* follows completion of Okazaki fragment maturation is yet to be explored. A final question regarding RFC complex specificities relates to their function in the DNA replication checkpoint. Three RFC complexes make additive contributions to checkpoint signaling in response to replication fork stalling (Bellaoui et al., 2003, Ben-Aroya et al., 2003, Kanellis et al., 2003). Whether an intricate choreography of PCNA loading and unloading is important for the replication checkpoint or whether checkpoint function is independent from PCNA loading and unloading is not known.

### Replisome-Coupled Events that Complete Chromosome Replication

In human cells, the MCM helicase has been proposed as a replication fork receptor for the Esco2 cohesin acetyl transferase (Ivanov et al., 2018, Minamino et al., 2018). The MCM helicase is among the first replisome components to encounter cohesin. Cohesin acetylation at this time would stabilize cohesin on DNA and impede any further DNA entry or exit. This could establish sister chromatid cohesion if the replisome was able to pass through cohesin rings. In contrast, budding yeast Eco1 appears to act where Okazaki fragment maturation takes place behind the replication fork. At this location, Eco1 might be positioned favorably to acetylate cohesin that has undergone renewed loading to embrace both sister DNAs (Lengronne et al., 2006, Murayama et al., 2018). We imagine that both replisome passage through cohesin rings and renewed cohesin loading behind the fork cooperate during cohesion establishment. The relative emphasis between the two pathways may differ between organisms. A very recent study emphasizes that human Esco2 also relies on PCNA interaction for its function (Bender et al., 2020).

*De novo* nucleosome deposition following DNA replication depends on the CAF-1 chromatin assembly complex that interacts with PCNA (Shibahara and Stillman, 1999). PCNA left behind following Okazaki fragment synthesis is thought to recruit CAF-1. This scenario fails to explain how CAF-1 promotes chromatin assembly on the leading strand. Our observation that Ctf18-RFC adds PCNA to the leading strands could explain how CAF-1 is recruited to both strands. Budding yeast telomere chromatin defects observed in the absence of Ctf18 could be a consequence of this a role (Hiraga et al., 2006). It will be interesting to investigate whether other post-replicative processes, e.g., replication-coupled DNA repair, also make use of the balanced PCNA distribution that Ctf18-RFC helps to create.

## STAR★Methods

### Key Resources Table

**Table:** 

REAGENT or RESOURCE	SOURCE	IDENTIFIER
**Antibodies**		

Mouse monoclonal anti-Smc3Ac	Gift from Shirahige Lab	N/A
Mouse monoclonal anti-Smc3 (361 F3C6, budding yeast)	Gift from Shirahige Lab	N/A
Mouse monoclonal anti-PCNA (5E6/2)	Cell Services Science Technology Platform, The Francis Crick Institute	N/A
Rabbit polyclonal anti-PCNA (Ab871, for eSPAN)	Gift from Zhang Lab	N/A
Rabbit polyclonal anti-V5	Abcam	ab15828
Rabbit polyclonal anti-HA	Abcam	ab9110
Rabbit polyclonal anti-Rad53	Abcam	ab104232
Mouse monoclonal anti-HA (12CA5)	Cell Services Science Technology Platform, The Francis Crick Institute	N/A
Mouse monoclonal anti-FLAG (M2)	Merck	Cat# F3165
Mouse monoclonal anti- α-tubulin (TAT-1)	Cell Services Science Technology Platform, The Francis Crick Institute	N/A
Mouse monoclonal anti-HIS (6G2A9)	Genscript	Cat# A00186
Anti-mouse IgG (HRP-conjugated)	GE Healthcare	Cat# NA931
Anti-rabbit IgG (HRP-conjugated)	GE Healthcare	Cat# NA934

**Chemicals, Peptides, and Recombinant Proteins**		

α-factor	Peptide Chemistry Science Technology Platform, The Francis Crick institute	N/A
Nocodazole	Merck	Cat# M1404
Hydroxyurea	Merck	Cat# H8627
Indole-3-acetic acid (IAA)	Merck	Cat# I3750
5-Bromo-2’-Deoxyuridine	Merck	Cat# B5002
G418	Merck	Cat# G8618
Formaldehyde solution	Merck	Cat# 252549
Paraformaldehyde	Merck	Cat# P6148
Phenylmethylsulfonyl fluoride (PMSF)	Merck	Cat# P7626
Pefabloc SC	Roche	Cat# 11 429 876 001
cOmplete EDTA-Free Protease Inhibitor Cocktail	Merck	Cat# 04693132001
Benzamidine	Merck	Cat# 12072
Bacitracin	Merck	Cat# B0125
Proteinase K	ThermoFisher	Cat# EO0491
Leupeptin	Generon	Cat# 51867.02
Benzonase Nuclease	Merck	Cat# E1014
RNase A	Merck	Cat# 10109169001
Protein Assay Dye	Bio-Rad	Cat# 5000006
Propidium iodide solution	Merck	Cat# P4864
GelRed Nucleic Acid Gel Stain	Biotium	Cat# 41003-1
ApaI	New England Biolabs	Cat# R0114S
APE1	New England Biolabs	Cat# M0282S
BsaBI	New England Biolabs	Cat# R0537L
tRNA (Bacterial, MRE600)	Merck	Cat# 10109541001
Chelex 100 Resin	Bio-rad	Cat# 1421253
Uracil DNA Glycosylase	ThermoFisher	Cat# EN0361
Recombinant Terminal Deoxynucleotidyl Transferase	ThermoFisher	Cat#10533065
Biotin-11-dXTP Tetralithium Salt	Affymetrix	Cat# 79015
SSPE	VWR International	Cat# J61214.K2
Imidazole	Merck	Cat# 68268
TCEP	Fluorochem Limited	Cat# M02624
*SIGMAFAST*™ Protease Inhibitor Tablets	Merck	Cat# S8820
Acetyl CoA	Merck	Cat# A2181

**Critical Commercial Assays**		

GeneChip™ S. cerevisiae Tiling 1.0R Array	ThermoFisher	Cat# 900645
HisTrap™ Fast Flow Crude	Merck	Cat# GE29-0486-31
Protein G Sepharose Beads Fast Flow	GE	Cat# 17-0618-01
Protein A Dynabeads	ThermoFisher	Cat# 10002D
ECL Prime Western Blotting Detection Regent	GE Healthcare	Cat# RPN2232
Q5 Site-Directed Mutagenesis Kit	New England Biolabs	Cat# E05545
InFusion HD cloning kit	Clontech Laboratories	Cat# 639634
CloneAmp HiFi PCR Premix	Clontech Laboratories	Cat# 639298
Whole Genome Amplification 2 Kit	Merck	Cat# WGA2
Agencourt AMPure XP Beads	Beckman-Coulter	Cat# A63881
Accel-NGS 1S Plus DNA Library Kit	Swift Biosciences	Cat# 10096
Accel-NGS 1S Unique Dual Indexing Kit	Swift Biosciences	Cat# 19096
ExoSAP-IT Express PCR Product Cleanup Reagent	VWR International	Cat# 75001
PowerUp SYBR Green Master Mix	ThermoFisher	Cat# A25742

**Experimental Models: Organisms/Strains**		

All Saccharomyces cerevisiae strains used in this study are listed in Table S1	Lab stock and this study	N/A
*Escherichia coli* DH5α competent cells	New England Biolabs	Cat# C2987U

**Oligonucleotides**		

All oligonucleotides used for qPCR are listed in Table S2	N/A	N/A

**Software and Algorithms**		

Snapgene v2.6	GSL Biotech	N/A
FlowJo v10.1	FlowJo	N/A
ImageQuant TL v8.1	GE Healthcare	N/A
ImageJ v1.50c	NIH, USA	N/A

**Deposited Data**		

ChIP chip and eSPAN sequencing data	GEO Accession Number GSE138056	N/A
Unprocessed gel images	https://data.mendeley.com/datasets/sh2wjmfvfh/draft?a=27586cf4-2026-483f-bcf0-2446ff5ef228	N/A

### Lead Contact and Materials Availability

Further information and requests for resources and reagents should be directed to and will be fulfilled by the Lead Contact, FU (frank.uhlmann@crick.ac.uk). All reagents generated in this study are available from the Lead Contact without restriction.

### Experimental Model and Subject Details

All *Saccharomyces cerevisiae* yeast strains used in this study were of the W303 background and are listed in Table S1.

### Method Details

#### Yeast Strains and Culture

Cells were cultured at 25°C in YPD medium, if not indicated otherwise. α-factor was used at a concentration of 7.5 μg/mL, nocodazole at 6 μg/mL and indole-3-acetic acid (IAA) acid at 88 μg/mL.

To monitor progression through a single cell cycle, cells were synchronized in G1 using α-factor for 2 h 45 min, and released into YPD to resume cell cycle progression. As soon as cells visibly budded, α-factor was added back to the culture to re-arrest cells in G1 following completion of cell division. To arrest cells in early S phase, G1 synchronized cells were released into YPD medium supplemented with 0.2 M hydroxyurea for 40 min.

Epitope tagging of endogenous genes and gene deletions were performed by gene targeting using polymerase chain reaction (PCR) products. To conditionally deplete Rfc1 or Cdc9, their C termini were fused to an auxin-inducible degron (Nishimura et al., 2009). Indole-3-acetic acid (IAA) was added to induce depletion for 2 h before release of α-factor synchronized cells from G1. To deplete Eco1, the *ECO1* promoter was replaced with the methionine-repressible *MET3* promoter and the C terminus was fused to an auxin degron. Cells were grown in SC medium lacking methionine and Eco1 depletion was achieved by shift to YPD medium containing 2 mM methionine as well as auxin. The Ctf18 ATPase mutations were created by PCR amplifying and TA cloning of a Pk epitope tagged genomic *CTF18* locus including an adjacent *LEU2* selection marker and flanking sequences. This was followed by site directed mutagenesis of the *CTF18* gene using the Q5 Site-Directed Mutagenesis Kit (New England Biolabs). PCR products containing the mutations were then amplified and integrated again at a previously unmodified *CTF18* locus. Pol2^EDD^ and Dcc1^KRR^ were created by cloning *DCC1* or the first 1650 bp of *POL2* under their own promoters into the yeast/*E. coli* shuttle vectors YIplac204 and YIplac128, respectively, followed by site directed mutagenesis using the above method. The plasmids contained a 5′ upstream region of the genes cloned behind the genes. The resulting plasmids were then linearized between gene and 5′ upstream region for gene replacement at the *POL2* and *DCC1* loci respectively.

#### Yeast Molecular Biology Techniques

##### Immunoblotting

Protein extracts for immunoblotting were prepared following cell fixation using trichloroacetic acid and separated by SDS-polyacrylamide gel electrophoresis before transfer to nitrocellulose membranes. Antibodies used for detection are listed in the Key Resources Table and were visualized using ECL reagents, via film (GE Healthcare) or with the Amersham Imager 600 (GE Healthcare). Quantification of band intensities was performed using ImageJ.

##### FACS analysis of DNA content

Cells were fixed in cold 70% ethanol for at least one h, then treated with 0.1 mg/mL RNase A in RNase buffer (50 mM Tris-HCl pH 7.5) at 37°C overnight. DNA was stained with 50 μg/mL propidium iodide in FACS buffer (200 mM Tris-HCl pH 7.5, 211 mM NaCl, 78 mM MgCl_2_). Samples were sonicated and diluted in 50 mM Tris-HCl pH 7.5. 10,000 cells per sample were analyzed using a FACSCalibur cell analyzer (BD Biosciences) and the data files were curated using FlowJo 10.

##### Protein interaction analysis

Cell extracts were prepared in co-immunoprecipitation buffer (50 mM Tris-HCl pH 7.5, 150 mM NaCl, 0.1% Triton X-100, protease inhibitors and benzonase) using glass beads breakage in a cooled Multi-Beads Shocker (Yasui Kikai). Extracts were cleared by centrifugation, precleared and incubated with either IgG coated Dynabeads (ThermoFisher) for Protein A pulldown or with Protein A Dynabeads previously ligated to the respective epitope-specific antibody. Beads were extensively washed and elution was carried out in SDS-PAGE loading buffer.

##### Eco1-PCNA interaction analysis

His_6_-Eco1 and His_6_-Eco1^-pip^ were expressed in *E. coli* BL21(DE3) pLysS for 18 h at 19°C after induction with 0.5 mM IPTG. Cells were collected by centrifugation, resuspended in Eco1 buffer (50 mM HEPES/NaOH pH 7.5, 250 mM NaCl, 150 μM acetyl-CoA, 40 mM imidazole, 10% Glycerol, 0.5 mM TCEP, 1 mM AEBSF, SigmaFast protease inhibitors) and lysed with a French press. Proteins were bound onto Histrap FF Crude (GE Healthcare) and eluted in Eco1 buffer containing 150 mM imidazole. Peak fractions were further purified over a HiLoad 16/600 Superdex 75 pg column (GE Healthcare) in Eco1 buffer without imidazole. Purified His-tagged proteins in PD Buffer (50 mM HEPES pH 7.5, 250 mM NaCl, 150 μM acetyl-CoA, 20 mM imidazole, 10% glycerol, 0.5 mM TCEP, 1 mM AEBSF, SigmaFast protease inhibitors) were first absorbed onto Nickel Sepharose beads (GE Healthcare). The beads were further incubated with yeast whole cell extracts and washed with PD Buffer containing 40 mM imidazole. Bound proteins were eluted in the PD Buffer containing 150 mM imidazole.

##### Chromatin immunoprecipitation analyses

Chromatin immunoprecipitation was performed as previously described (Katou et al., 2006). Briefly, cells were fixed with formaldehyde and harvested. Protein extracts were prepared and chromatin disrupted by sonication. DNA fragments cross-linked to the protein of interest were enriched by immunoprecipitation. After reversal of cross-links, DNA both from immunoprecipitates and from whole cell extract was purified and quantified using the PowerUP SYBR Green Master Mix (ThermoFisher) and a Viia7 Real-Time PCR System (Thermo Fisher). All primer sequences used are listed in Table S2. Microarray analyses to visualize chromosomal distribution patterns were performed as previously described (Lengronne et al., 2004), with the following modification. Library preparation and amplification were carried out using the GenomePlex Complete Whole Genome Amplification 2 kit (Sigma). 4 - 7 μg of amplified DNA were fragmented using human apurinic/apyrimidinic endonuclease (APE1) in the presence of uracil DNA glycosylase, and then labeled with Biotin-11-dXTPs using recombinant terminal deoxynucleotide transferase before hybridization to Affymetrix GeneChip Yeast Genome 2.0 arrays (Merck).

##### BrdU-IP microarray analysis

For the BrdU-IP microarray analysis, oligonucleotide probe signals were grouped in 200 bp bins. Signal intensities were blotted relative to their respective whole genome DNA input samples. 52 early firing origins were manually selected based on robustness of the signal and separation from neighboring peaks. The median maximal intensities of the BrdU peaks was derived for each strain at each time point and normalized to 1. A thresholding value was set at 0, and the width of the selected BrdU peaks was analyzed.

##### Sister chromatid cohesion assay

Cells carrying a GFP-marked *URA3* locus (Michaelis et al., 1997) were synchronized in G1 using α-factor and released into a nocodazole-imposed mitotic arrest. Cells were fixed with ice-cold 100% ethanol for at least two h, mounted on thin agarose patches and imaged using an Axioplan 2 fluorescence microscope (Zeiss). The cohesion status of the URA3 locus was scored in at least 100 cells per sample.

##### Enrichment and sequencing of protein-associated nascent DNA (eSPAN)

eSPAN was performed as described (Yu et al., 2018). Briefly, cells were synchronized in G1 using α-factor and released into YPD medium supplemented with BrdU. Samples were taken either at predetermined times in early S phase, or following release into HU-containing medium. Aliquots were fixed with formaldehyde and nuclear extracts prepared. Protein pulldown was performed using the fast-ChIP protocol (Nelson et al., 2006). The purified ChIP DNA was heat denatured and subjected to a second round of BrdU pulldown (eSPAN). In parallel, input DNA was also subjected to BrdU-IP. This total BrdU DNA, the protein ChIP fraction and the recovered eSPAN DNA were extensively purified. Quality control of the purified DNA was performed by real-time quantitative DNA using oligos targeting active and inactive origins. Strand specific library preparation was then performed using the Accel-NGS 1S Plus DNA Library Kit (Swift Biosciences).

##### eSPAN data analysis

101bp paired-end sequencing of the strand specific libraries representing input DNA, BrdU-IP DNA, ChIP DNA and eSPAN DNA was performed on the Illumina HiSeq 2500 or 4000 platforms. Raw reads from each sample were adaptor-trimmed using cutadapt (version 1.9.1) (Martin, 2011) with parameters “-a AGATCGGAAGAGC -A AGATCGGAAGAGC–minimum-length=25 –quality-cutoff=20.” As a result of the protocol used for the library preparation, a second round of trimming was performed using cutadapt with the parameter “-U 10” to remove the last 10bp of read 2. BWA (version 0.6.2) (Li and Durbin, 2009) with default parameters was used to perform genome-wide mapping of the trimmed reads to the yeast sacCer3 genome assembly downloaded from the UCSC (Karolchik et al., 2004). SAMtools (version 1.3.1) (Li et al., 2009) and BamTools (version 2.4.0) (Barnett et al., 2011) were used to filter the alignments to only include uniquely mapped reads with insert size ≤ 2kb, and ≤ 4 mismatches in either read.

Alignments were split by strand using a custom script and normalized bedGraph coverage tracks were generated representing the signal per million mapped paired-reads using BEDTools genomeCoverageBed (version 2.26.0) (Quinlan and Hall, 2010) with the parameters “-bg -pc -du -strand <STRAND> -scale <SCALE_FACTOR>.” BedGraph files were converted to bigWig using the wigToBigWig binary available from the UCSC with the “-clip” parameter (Kent et al., 2010).

The computeMatrix reference-point command from the deepTools package (version 2.5.3) (Ramírez et al., 2016) was used to generate coverage matrices with respect to the given set of ARS intervals. The parameters used were “–referencePoint center–upstream 20000–downstream 20000–binSize 100–averageTypeBins mean –missingDataAsZero–scale 1.” Meta-profile plots were generated with the ggplot2 package (version 2.2.1) within the R programming environment (version 3.3.1).

### Quantification and Statistical Analysis

All experiments from which quantitative results were obtained were repeated three times on separate occasions. For a quantitative assessment of sister chromatid cohesion, at least 100 cells were scored at each time point in each replicate. Quantification of western blot signals was performed using chemiluminescence and the Amersham Imager 600 (GE Healthcare). Band intensities were then analyzed using ImageJ. Quantitative analysis of chromatin immunoprecipitates was performed using the PowerUP SYBR Green Master Mix and a Viia7 Real-Time PCR System (Thermo Fisher). Means and standard errors from the three biological repeats are shown in all cases.

### Data and Code Availability

The sequencing data generated in this study has been deposited with the Gene Expression Omnibus https://www.ncbi.nlm.nih.gov/geo/ with the accession number GSE138056. Unprocessed gel images presented in this manuscript can be found at https://data.mendeley.com/datasets/sh2wjmfvfh/draft?a=27586cf4-2026-483f-bcf0-2446ff5ef228.

## Supporting Citations

The following reference appears in the Supplemental Information: Bouchoux and Uhlmann, 2011.

## Supplemental Information

Document S1. Figures S1–S7

Table S1. Yeast Strains Used in this Study, Related to STAR Methods

Table S2. Primers Used for Quantitative Real-Time PCR, Related to STAR Methods

Document S2. Article plus Supplemental Information
