# Supplementary material for: Division of Labor between PCNA Loaders in DNA Replication and Sister Chromatid Cohesion Establishment
Source: Mol Cell. 2020 May 21;78(4):725–738.e4. doi: 10.1016/j.molcel.2020.03.017 (PMC7242910; doi:10.1016/j.molcel.2020.03.017)
Supplement: Document S1. Figures S1–S7 [file mmc1.pdf]

Molecular Cell, Volume *78*

Supplemental Information

Division of Labor between PCNA Loaders in DNA Replication and Sister Chromatid Cohesion Establishment

Hon Wing Liu, Céline Bouchoux, Mélanie Panarotto, Yasutaka Kakui, Harshil Patel, and Frank Uhlmann

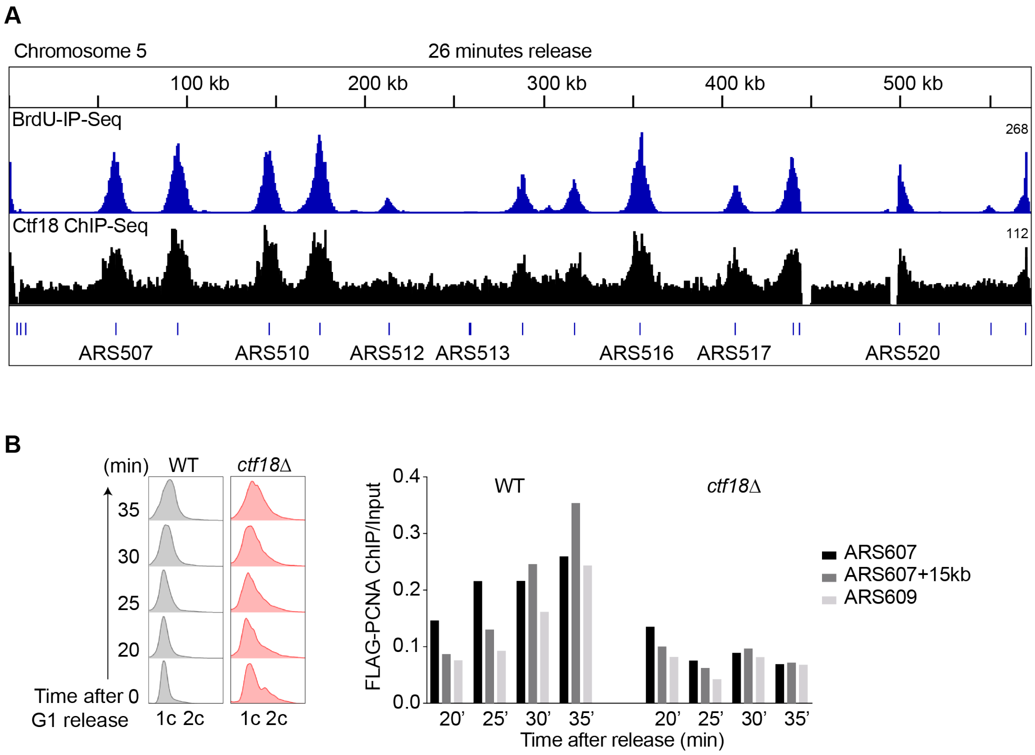

Figure S1. Ctf18 Is a PCNA Loader during Undisturbed S Phase, Related to Figure 1

(A) Ctf18 localizes to replication forks during undisturbed S phase. Cells were synchronized in G1 by α-factor treatment and released into BrdU-containing medium for 26 minutes before cell fixation and crosslinking. BrdU-IP and Ctf18 ChIP were performed and recovered DNA was analyzed by sequencing. BrdU and Ctf18 distributions are depicted along the indicated stretch of chromosome 5. The specified coverage was obtained by treating each pair of reads as a single fragment and normalizing the total number of mapped reads to 1 million.

(B) Ctf18 deletion results in reduced PCNA levels at synchronously progressing DNA replication forks. G1-arrested wild type (WT) or *ctf18Δ* cells were released into synchronous cell cycle progression. Cells were harvested at the indicated times and processed for FLAG-PCNA ChIP. Cell cycle progression was monitored by FACS analysis of DNA content. PCNA enrichment at the early origin (ARS607), a locus 15kb downstream (ARS607+15kb) and a late firing origin (ARS609) was assessed by quantitative real-time PCR.

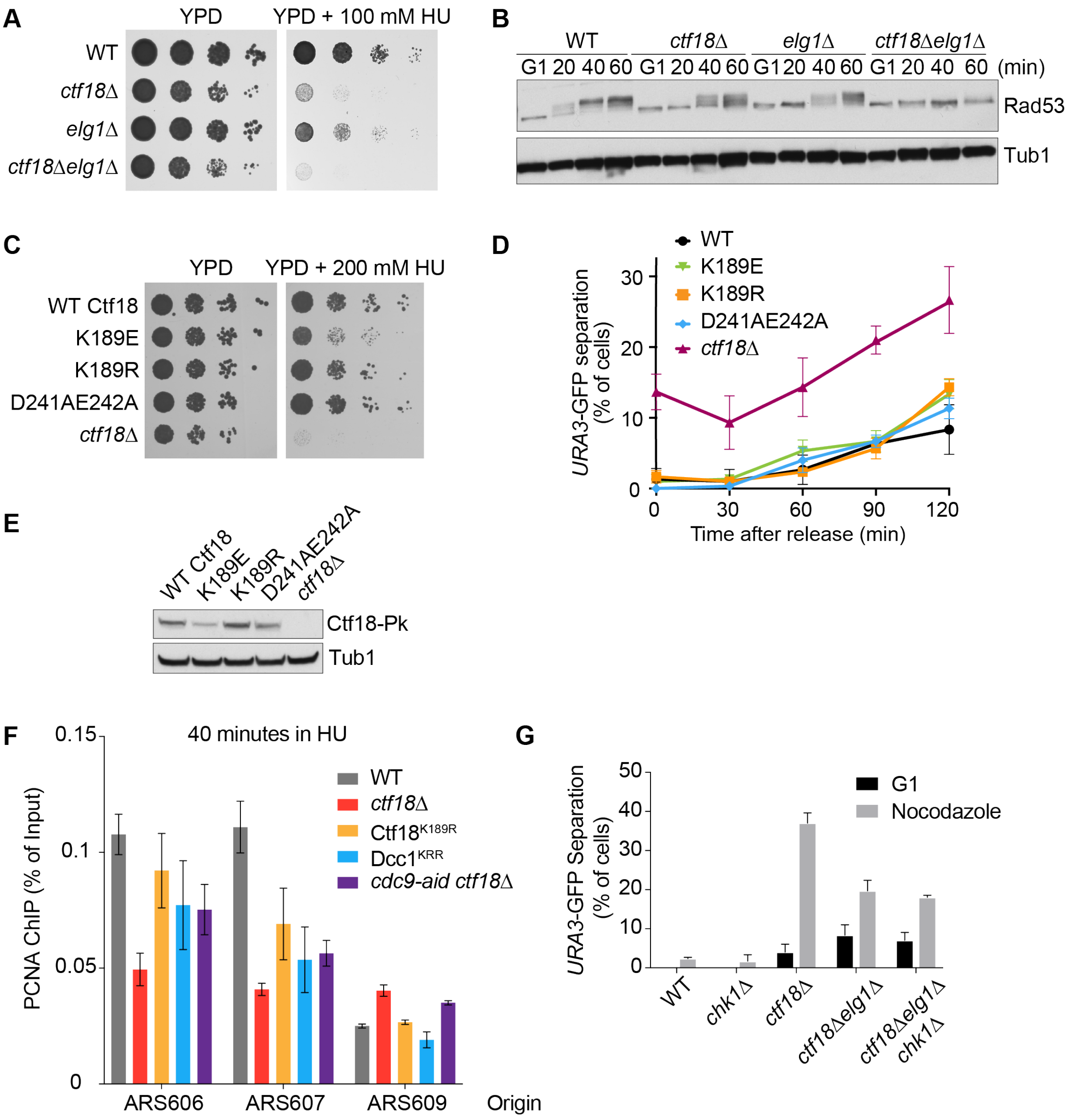

Figure S2. Separation of Cohesion Establishment and Replication Checkpoint Functions of Ctf18, Related to Figure 1

(A) Elg1 removal compounds the HU sensitivity in a *ctf18Δ* strain. Ten-fold serial dilutions of the indicated strains were spotted on YPD agar without or containing 100 mM HU.

(B) Elg1 and Ctf18 synergistically promote Rad53 phosphorylation upon replication fork stalling. Cells of the indicated genotypes were synchronized in G1 and released into HU-containing medium. Rad53 phosphorylation was monitored at the indicated time points by western blotting. Tub1 serves as a loading control.

(C) HU sensitivity of Ctf18 ATPase mutants. Ten-fold serial dilutions of the indicated strains were spotted on YPD agar without or supplemented with 200 mM HU.

(D) Ctf18 ATPase mutants exhibit unperturbed cohesion. Cells of the indicated genotypes were synchronized in G1 and released into nocodazole-containing medium for mitotic arrest. Sister chromatid cohesion was assessed at the GFP-marked *URA3* locus at indicated time points. Means ± SE of three independent experiments are shown.

(E) Western blot to compare Ctf18-Pk levels in asynchronous cultures of the indicated Ctf18 ATPase mutants. Tub1 serves as a loading control.

(F) PCNA levels at replication forks under various conditions. Cells of the indicated genotypes were synchronized in G1 by α-factor treatment and released into HU-containing medium for an early S phase arrest. PCNA enrichment close to early (ARS606 and 607) and a late firing (ARS609) replication origin were quantified by real-time PCR. Means ± SE from three independent experiments are shown.

(G) Rescue of the *ctf18Δ* cohesion defect by Elg1 removal is not due to DNA damage-induced cohesion. Cells of the indicated genotypes were synchronized in G1 by α-factor treatment and released into nocodazole-containing medium for 120 minutes to achieve a mitotic arrest. Sister chromatid cohesion was assessed at the GFP-marked *URA3* locus at the indicated times. Means ± SE of three independent experiments are shown.

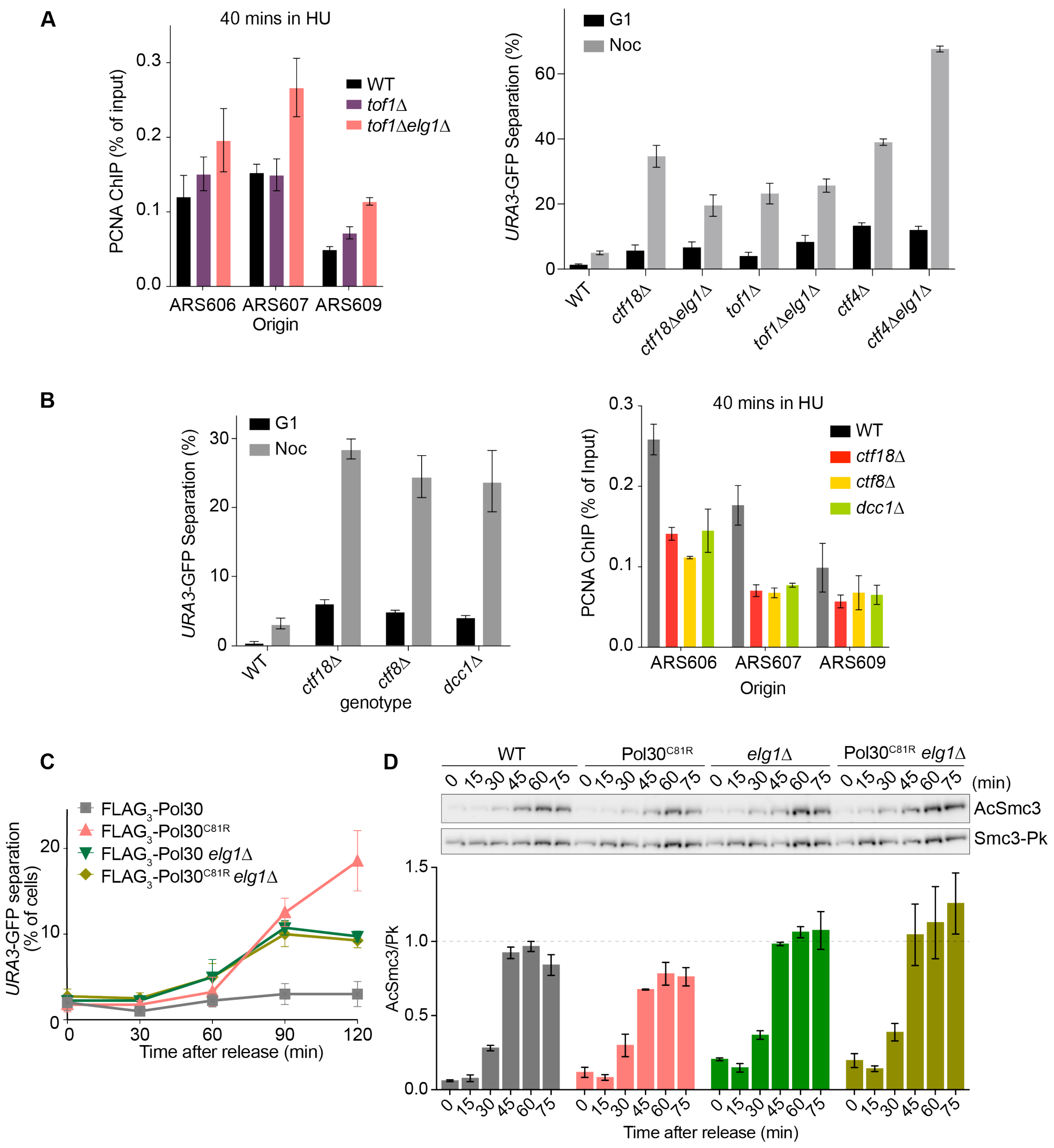

Figure S3. Further Analyses on the Role of PCNA in Cohesion Establishment, Related to Figure 2
(A) *elg1* deletion rescues sister chromatid cohesion in the absence of Ctf18, but not of other cohesion establishment factors, Tof1 or Ctf4. Cells of the indicated genotypes were synchronized in G1 by α-factor treatment, released into HU containing medium for arrest in early S-phase for PCNA ChIP analysis or released into nocodazole-containing medium to achieve a mitotic arrest to assess sister chromatid cohesion. Means ± SE of three independent experiments are shown.
(B) Ctf8 and Dcc1 make equal contributions to those of Ctf18 during PCNA loading and sister chromatid cohesion establishment. Sister chromatid cohesion and PCNA levels in cells of the indicated genotypes were assessed as in (A).
(C) Destabilizing PCNA results in a cohesion defect, rescued in the absence of Elg1. Cells of the indicated genotypes were synchronized in G1 and released into nocodazole-containing medium for mitotic arrest. Sister chromatid cohesion was assessed at the GFP-marked *URA3* locus at indicated time points. Means ± SE of three independent experiments are shown.
(D) as (C) but Smc3 acetylation was quantified relative to total Smc3 levels. Means ± SE from three independent experiments are shown.

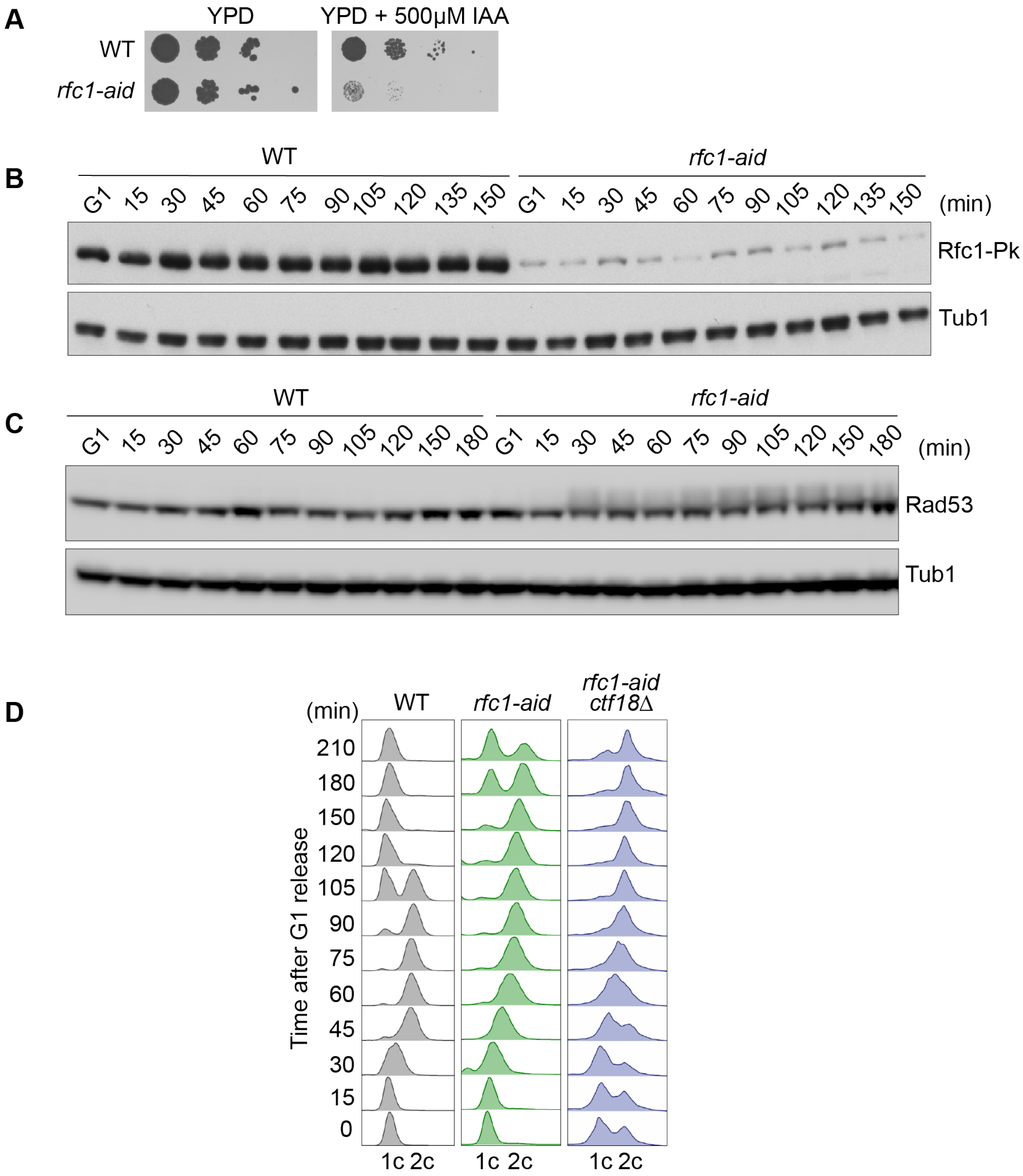

Figure S4. Further Characterization of *rfc1-aid* Cells, Related to Figure 3
(A) Rfc1 depletion impedes cell growth. Ten-fold serial dilutions of the indicated strains were spotted on YPD plates without or supplemented with 500 μM auxin (IAA).
(B) Reduced Rfc1 protein levels upon auxin-induced depletion. Aliquots of cells in the experiment shown in Figure 3A were harvested at the indicated times and processed for western blotting. Rfc1 levels were monitored using an antibody against a C-terminal Pk epitope tag. Tubulin serves as a loading control.
(C) Replication checkpoint activation due to Rfc1 depletion. The indicated strains were arrested into G1 and released into synchronous cell cycle progression. At the indicated times, cells were harvested and protein extracts prepared for western blotting. Rad53 phosphorylation, indicative of replication checkpoint activation, was monitored. Tub1 served as a loading control.
(D) Absence of Ctf18 does not further impede DNA replication in Rfc1-depleted cells. Cells of the indicated genotypes were synchronized in G1 and released to pass through a synchronous cell cycle before re-arrest in the following G1. FACS analysis of DNA content was performed to monitor DNA replication and cell division.

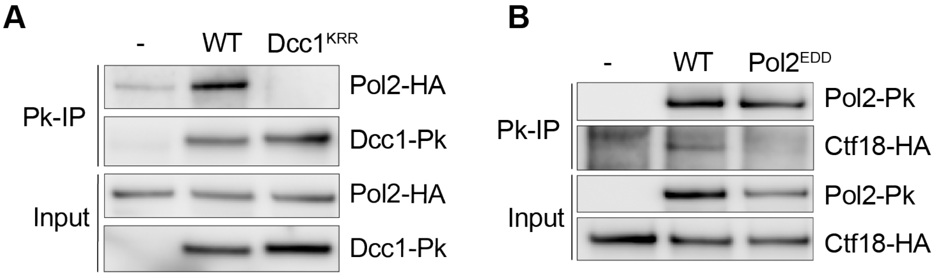

Figure S5. Disruption of the Dcc1-Pol2 Interaction, Related to Figure 4
(A) Wild type Dcc1, but not Dcc1^{KRR}, interacts with Pol2. Asynchronous cultures of the indicated strains were harvested, cell extracts prepared and Pk epitope-tagged Dcc1 was immunoprecipitated. Coprecipitation of Pol2 was analyzed by western blotting.
(B) Wild type Pol2, but not Pol2^{EDD}, interacts with Ctf18-RFC. As (A), but coprecipitation of Ctf18 with Pol2 or Pol2^{EDD} was analyzed.

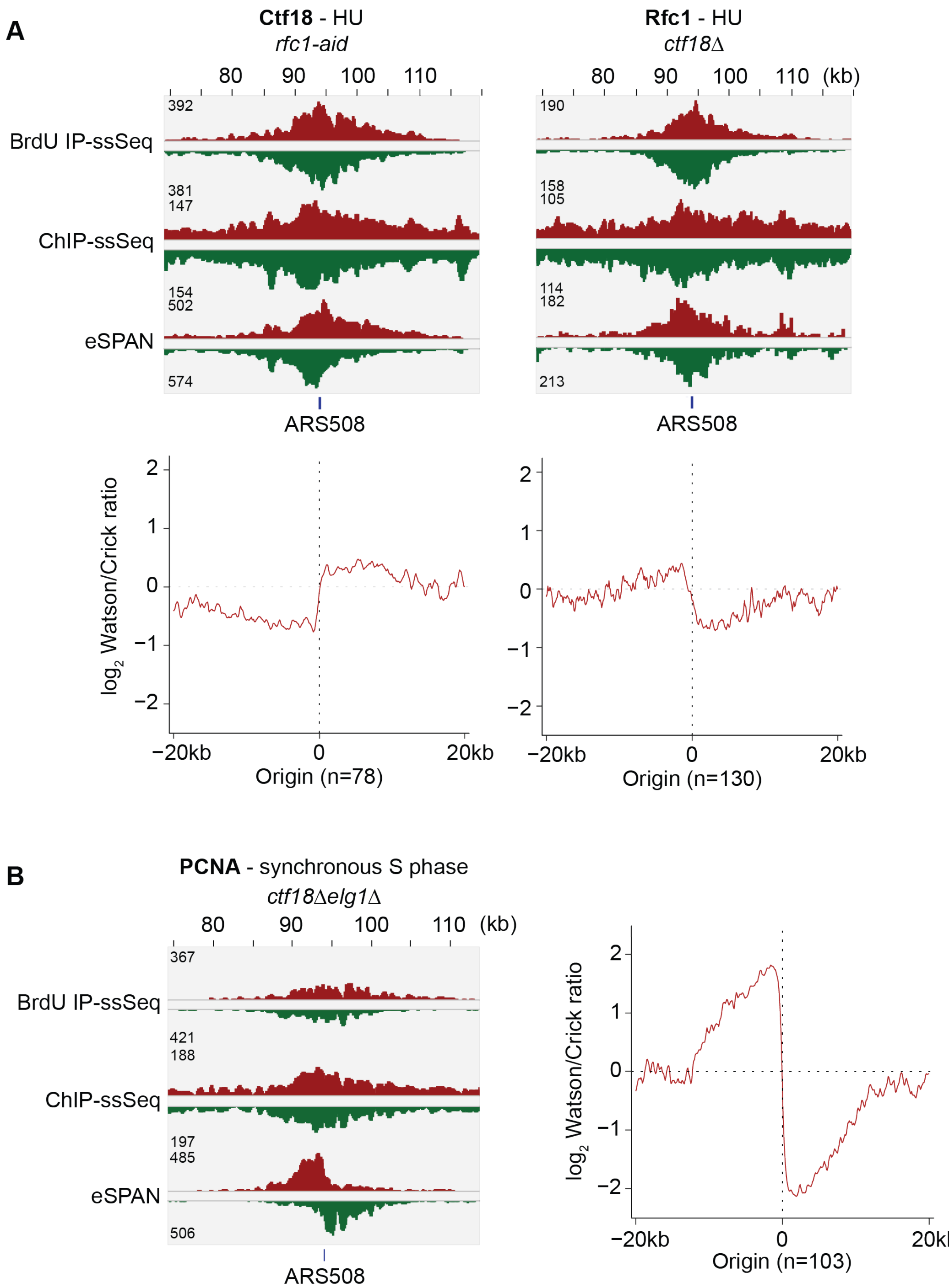

Figure S6. Additional eSPAN Analyses, Related to Figure 6
(A) Ctf18 and Rfc1 do not replace each other. Cultures of *ctf18Δ* cells harboring Rfc1-Pk, and *rfc1-aid* cells harboring Ctf18-HA were synchronized and processed as in Figure 5B. Upper panels: BrdU-IP ssSeq, ChIP ssSeq and eSPAN profiles for both proteins are depicted surrounding an early origin ARS508. Watson (red) and Crick (green) coverage were calculated by treating each read pair as a single fragment and normalizing the total number of mapped reads to 1 million. Lower panels: Watson/Crick ratio metaprofiles, normalized to BrdU-IP ssSeq signals, centered on 78 (*rfc1-aid* dataset) and 130 (*ctf18Δ* dataset) pooled origins.
(B) Elg1 removal in a *ctf18Δ* background leads to lagging strand PCNA accumulation. PCNA eSPAN was performed in an *ctf18Δ elg1Δ* strain as in Figure 6.

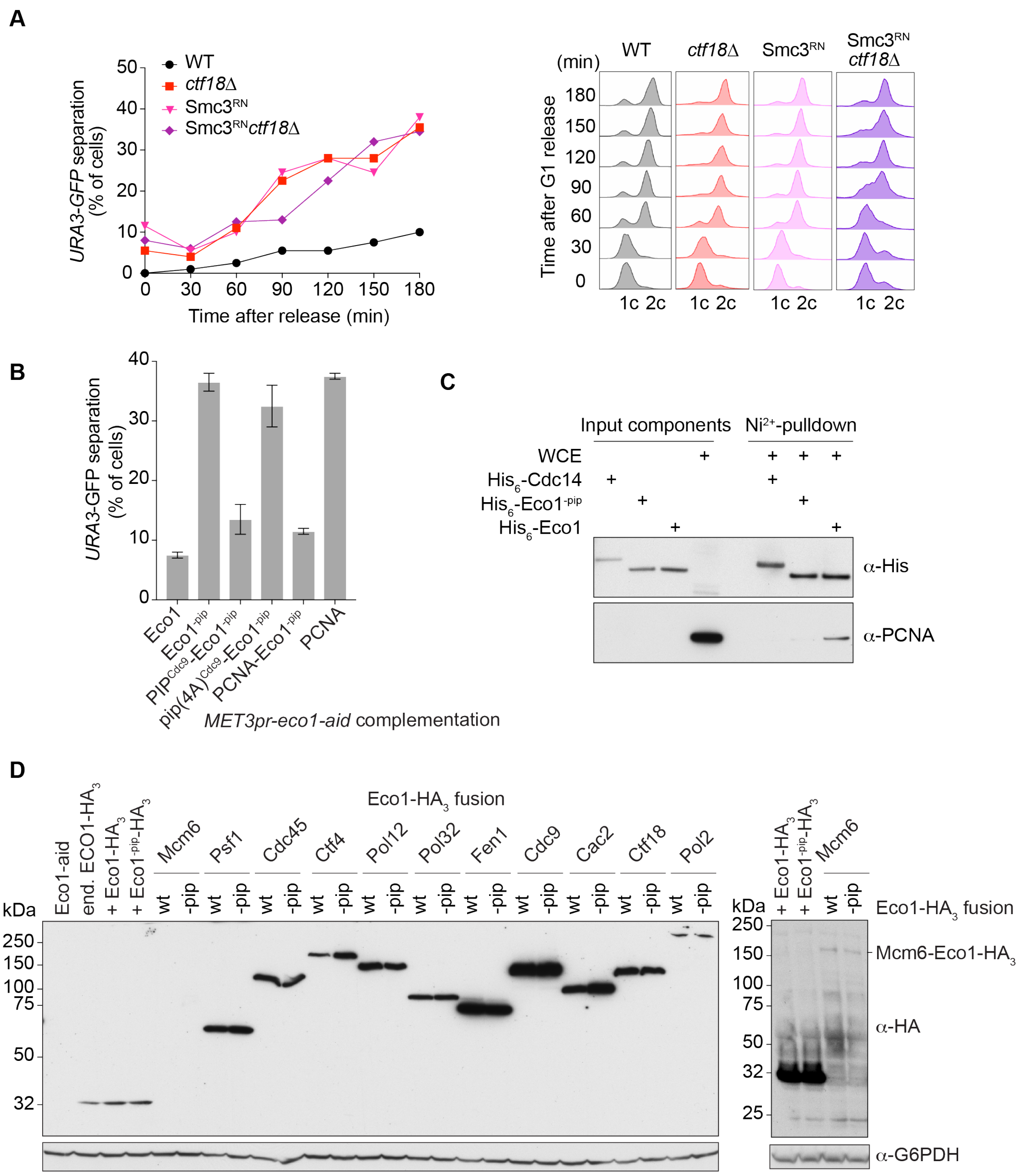

Figure S7. Ctf18 Acts by Means of Cohesin Acetylation, Related to Figure 7

(A) Ctf18 acts in sister chromatid cohesion establishment via Smc3 acetylation. Cells of the indicated genotypes were synchronized in G1 and released into nocodazole containing medium for mitotic arrest. Cell cycle synchrony was confirmed by FACS analysis of DNA content. Sister chromatid cohesion was assessed at the GFP-marked *URA3* locus at the indicated time points. Means ± SE of three independent experiments are shown.

(B) Defective cohesion establishment in $Eco1^{-pip}$ cells is restored by functional PIP fusions. *MET3pr-eco1-aid* cells expressing the indicated additional proteins were synchronized in G1, depleted of endogenous Eco1, and released into nocodazole-containing medium for mitotic arrest. Sister chromatid cohesion was assessed at the GFP-marked *URA3* locus at indicated time points. Means ± SE of three independent experiments are shown.

(C) A PIP box dependent Eco1-PCNA interaction. Purified recombinant His_6-Cdc14 as a negative control (Bouchoux and Uhlmann, 2011), His_6-Eco1 and His_6-$Eco1^{-pip}$ were absorbed onto nickel Sepharose beads. Those were further incubated with yeast whole cell extracts, washed and eluted in with buffer containing 150 mM imidazole. Bound proteins were analyzed by western blotting.

(D) Expression of Eco1 and $Eco1^{-pip}$ fusion proteins with replisome components in budding yeast. Strains with the indicated fusion proteins were grown and samples harvested for western blot analysis to confirm fusion protein expression. The Mcm6-Eco1 and Mcm6-$Eco1^{-pip}$ fusion proteins were expressed at only low levels and required a longer exposure for detection. Note that the *ECO1* or *eco1^{-pip}* genes were fused with the respective endogenous gene loci. Therefore, despite the low levels, each Mcm6 protein that travels with the replication fork is associated with Eco1 or $Eco1^{-pip}$.
